# Transneuronal Transport of Ligand-Conjugated Upconversion Nanoparticles from the Peripheral to the Central Nervous System

**DOI:** 10.1021/jacsau.6c00949

**Published:** 2026-08-20

**Authors:** Haitao Liu, Hengde Li, Xi Chen, Saman Hamidi, Angelo H. ALL

**Affiliations:** † Department of Chemistry, 26679 Hong Kong Baptist University, Kowloon, Hong Kong SAR 999077, China; ‡ Morsani College of Medicine, 7831 University of South Florida, Tampa, Florida 33602, United States

**Keywords:** intra-axonal movement, neuronal retrograde transport, trans-synaptic translocation, functionalized nanomaterials, upconversion nanoparticles

## Abstract

Despite significant advancements in neuro-nanotechnology, the movement of nanomaterials between peripheral and central neurons for applications such as cargo delivery, imaging, and tracking remains a formidable challenge. This limitation highlights the critical necessity for developing engineered materials that can facilitate efficient intra-axonal transport and trans-synaptic translocation. In this study, we report the design and synthesis of core–shell–shell upconversion nanoparticles (CSS-UCNPs) featuring a NaYF_4_:YbEr core sequentially coated with active NaYF_4_:YbNd and inert NaYF_4_ shells. To achieve neuron-specific targeting, the architecture was surface-functionalized with poly­(ethylene glycol) (PEG)-maleimide and conjugated to the RVG29 peptide (CSS-PEG-RVG29). Characterization by transmission electron microscopy, dynamic light scattering, and spectroscopic analyses confirmed uniform particle size (42.9 nm radius) and efficient upconversion luminescence (λ_ex_ = 808 nm, λ_em_ = 500−575 nm). *In vitro* assays demonstrated low cytotoxicity in Neuro2a, NSC34, and C6 cell lines at concentrations up to 500 μg/mL, enhanced cellular uptake mediated by RVG29, and no detectable alterations in spinal neuronal action potential firing, as assessed by patch-clamp electrophysiology. *In vivo*, intrafascicular injection into the mouse sciatic nerve (2 mg/mL, 2 μL) resulted in nanoparticle transport to the dorsal part of the spinal cord, with localization in higher regions of the central nervous system (CNS) and cortices, as visualized by multiphoton microscopy. In contrast, control experiments utilizing scrambled RVG29-conjugated nanoparticles demonstrated negligible accumulation within the CNS. These findings establish CSS-PEG-RVG29 as a promising platform for peripheral-to-CNS cargo delivery, with broad potential applications.

## Graphical abstract

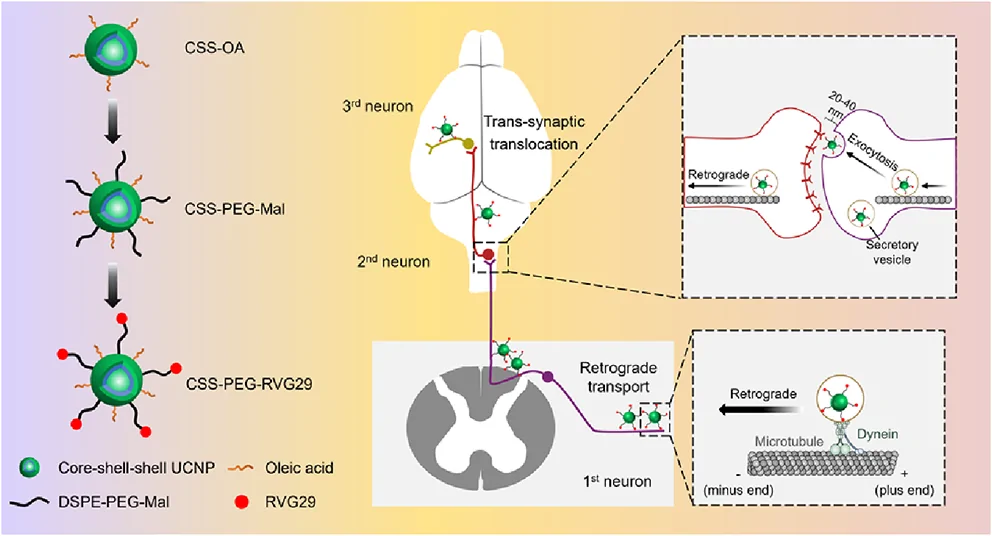

## 1 Introduction

Axonal retrograde transport refers to the process by which neuronal cargos are moved from distal axon terminals toward the soma along microtubule tracks, mediated by ATP-dependent motor proteins, such as dynein. A similar anterograde transport is mediated by kinesin motor protein.^1^ The transported materials include endocytosed signaling molecules, neurotrophic factors such as nerve growth factor^1^ and brain-derived neurotrophic factor,^2^ as well as organelles including lysosomes, endosomes, and damaged mitochondria.^3^ The intra-axonal transport is vital for maintaining neuronal homeostasis, facilitating long-distance trophic signaling, supporting synaptic remodeling,^4^ and activating gene expression programs necessary for injury response and repair.^5^ Notably, certain neurotropic viruses such as rabies virus,^6^ herpes simplex virus,^7^ and poliovirus,^8^ as well as certain viral toxins,^9,10^ exploit the structural and functional architecture of the nervous system to achieve central nervous system (CNS) entry. By utilizing retrograde axonal transport and undergoing trans-synaptic transmission from peripheral and spinal nerves, they can propagate through neural circuits and effectively bypass the blood–brain barrier. By leveraging retrograde and trans-synaptic transport pathways, biomimetic nanomaterials or molecular delivery systems designed to emulate viral entry mechanisms offer a promising strategy for minimally invasive and neuron-specific CNS delivery from the periphery.^11,12^

RVG29 is a 29-amino-acid peptide derived from the rabies virus glycoprotein that binds selectively to neuronal cells.^13^ The RVG29 derivative exploits the inherent neurotropism of the parent virus to achieve high-affinity binding to receptors, such as the nicotinic acetylcholine receptors (nAChRs) and γ-aminobutyric acid (GABA) receptors.^14^ Upon receptor binding, RVG29 triggers clathrin-mediated endocytosis,^15^ followed by intracellular trafficking that promotes endosomal escape and dynein-driven retrograde axonal transport.^16,17^

Upconversion nanoparticles (UCNPs) are a class of nanomaterials capable of converting low-energy near-infrared (NIR) excitation light^18–22^ into higher-energy visible light or ultraviolet emission through nonlinear optical processes.^23^ UCNPs are commonly composed of nanocrystals doped with rare-earth ions such as ytterbium (Yb^3+^), erbium (Er^3+^), or thulium (Tm^3+^).^24^ They can be synthesized by methods like high-temperature thermal decomposition or coprecipitation.^25^ Both high-temperature thermal decomposition and coprecipitation methods enable rigorous control over nanoparticle size, morphology, and photoluminescence profiles by modulating critical parameters, including dopant concentration, thermal conditions, solvent stoichiometry, and the incorporation of surface-stabilizing agents such as oleic acid, poly­(ethylene glycol) (PEG), or citrate.^26–28^ Their NIR excitation and multicolor visible emission effectively overcome the limitations of tissue scattering and autofluorescence encountered in conventional optical imaging, providing high-resolution, deep-tissue imaging^29,30^ with low background noise and superior photostability.^31,32^ These properties offer significant advantages for neuroscience research, particularly the deep-tissue imaging of neuronal populations. Advanced surface-engineering strategies, utilizing ligand–receptor molecular recognition, have been developed to optimize the bioavailability and neural-targeting efficiency of upconversion nanoplatforms.^33,34^

In this study, our main objective is to evaluate CSS-PEG-RVG29 nanoparticles as a tracer for targeted/enhanced neuronal uptake and long-distance transneuronal transport from the peripheral to the CNS. We provide a comprehensive characterization of the physicochemical profiles, biocompatibility, and neurotropic specificity of the CSS-PEG-RVG29 nanoparticles. Our results demonstrated their capacity for retrograde axonal transport and trans-synaptic propagation via intrafascicular administration into the mouse sciatic nerve. These findings suggest that RVG29-functionalized UCNPs provide a promising approach for CNS cargo delivery.

## 2 Results

### 2.1 Synthesis and Characterization of C, CS, and CSS-UCNPs

CSS structure UCNPs were synthesized via a high-temperature thermal decomposition approach. The core, composed of NaYF_4_:YbEr nanocrystals, served as the source of green luminescence. The intermediate shell consisted of NaYF_4_:YbNd, with Nd^3+^ functioning as an additional sensitizer to enhance brightness and facilitate excitation at 808 nm within the transparent NIR window. An NaYF_4_ inert shell was introduced to further improve the spectral properties. Transmission electron microscopy (TEM) images of the core (C), core–shell (CS), and core–shell–shell (CSS) structures are presented in Figure 1A. The C and CS nanoparticles exhibited spherical morphology, whereas the CSS nanoparticles were predominantly cylindrical. The CSS UCNPs exhibited high size uniformity, which is essential for consistent physical and biological performance and reproducible experimental outcomes. Hydrodynamic diameter measurements by dynamic light scattering (DLS) indicated that the radii of C, CS, and CSS particles in aqueous solutions were 20.7, 25.8, and 31.7 nm, respectively (Figure 1B). XRD data showed their crystalline structure and phase composition (Figure S2). Figure 1C shows the emission spectra of NaYF_4_:YbEr@NaYF_4_:YbNd@NaYF_4_ obtained under 808 nm laser excitation. In the absence of a sensitizer, NaYF_4_:YbEr fails to absorb the excitation light at 808 nm, resulting in no emission. However, when the core particles are coated with an active shell, green light emission is achieved. Three distinct luminescence peaks are observed at 520, 541, and 661 nm, corresponding to transitions of Er^3+^ ions ^2^H_11/2_ → ^4^I_15/2_, ^4^S_3/2_ → ^4^I_15/2_, and ^4^F_9/2_ → ^4^I_15/2_, respectively. The subsequent addition of an inert shell reduces surface defects and quenching effects, significantly enhancing the upconversion luminescence intensity (Figure 1C). The upconversion emission intensity *I*_out_ is proportional to the incident pump power *I*_exc_ (*I*_out_ ∝ (*I*_exc_)*^n^*). Here, *n* represents the number of infrared multiphotons involved in the generation of a single visible photon. The observed slope indicates that the green and red fluorescence emissions of CSS occur through a two-photon process. Additionally, the slopes of the double-logarithmic plots were determined to be 1.57 (541 nm), 1.58 (520 nm), and 1.94 (661 nm), with energy losses implied during the upconversion process, as indicated by n values of <2 (Figure S1).

**Figure 1:**
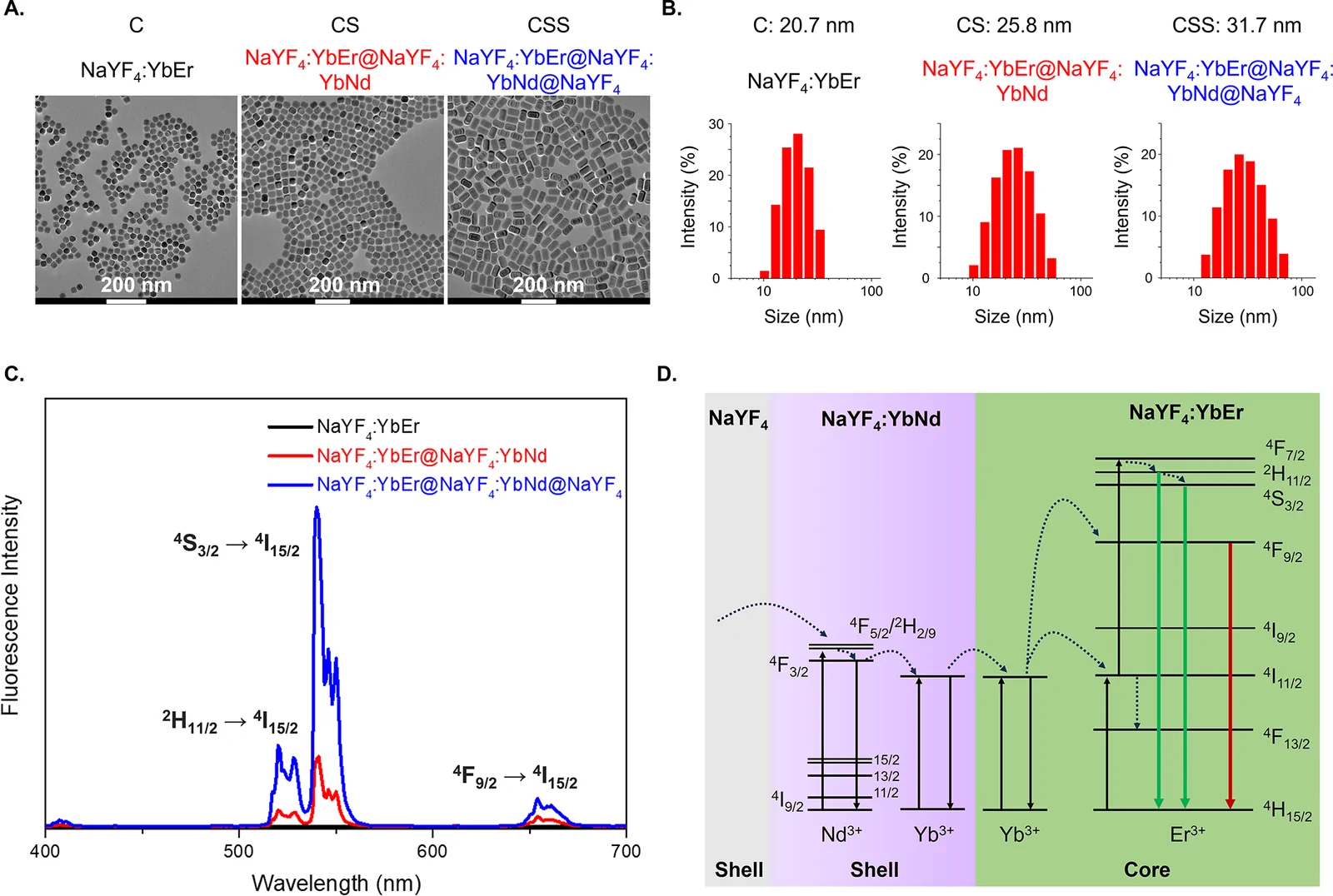
Characterization of C, CS, and CSS structure UCNPs: (A) TEM images show the morphology of three types of UCNPs, C, CS, and CSS; (B) three graphics show the radius of UCNPs measured through DLS; (C) emission spectrum of CSS under 808 nm laser excitation (1 mg/mL in cyclohexane, laser: 2.8 W/cm^2^, slit: 0.5 nm); and (D) proposed energy transfer upconversion mechanism in CSS UCNPs.

### 2.2 Synthesis and Characterization of CSS-PEG-Mal, CSS-PEG-RVG29, and CSS-PEG-scrRVG29

CSS-PEG-Mal nanoparticles were successfully prepared by encapsulating CSS with DSPE-PEG and DSPE-PEG-Mal through solvent evaporation, followed by repeated centrifugation to remove unencapsulated ligands. Subsequent surface functionalization was achieved via thiol-maleimide conjugation of RVG29 or scrambled RVG29 (scrRVG29) peptides, each containing a C-terminal cysteine, with CSS-PEG-Mal in an aqueous buffer (Figure 2A). The conjugation of both CSS-PEG-RVG29 and CSS-PEG-scrRVG29 was confirmed by IR spectroscopy (Figure 2B). This modular synthetic approach enables the generation of surface-engineered CSS nanocarriers with tunable targeting ligands, while the use of PEGylation imparts enhanced colloidal stability and biocompatibility in a physiological environment. In this work, the neurotropic peptide RVG29 was employed as a neuronal-targeting ligand, which could specifically bind to neuron-enriched nAChRs and GABA receptors, making it an ideal candidate for CNS delivery of theranostic agents.^35^ After conjugation, the hydrodynamic particle radius increased from 31.7 to 42.9 nm (Figure 2C). The nanoparticles exhibited an exceptional fluorescence emission in aqueous solution (Figure 2D).

**Figure 2:**
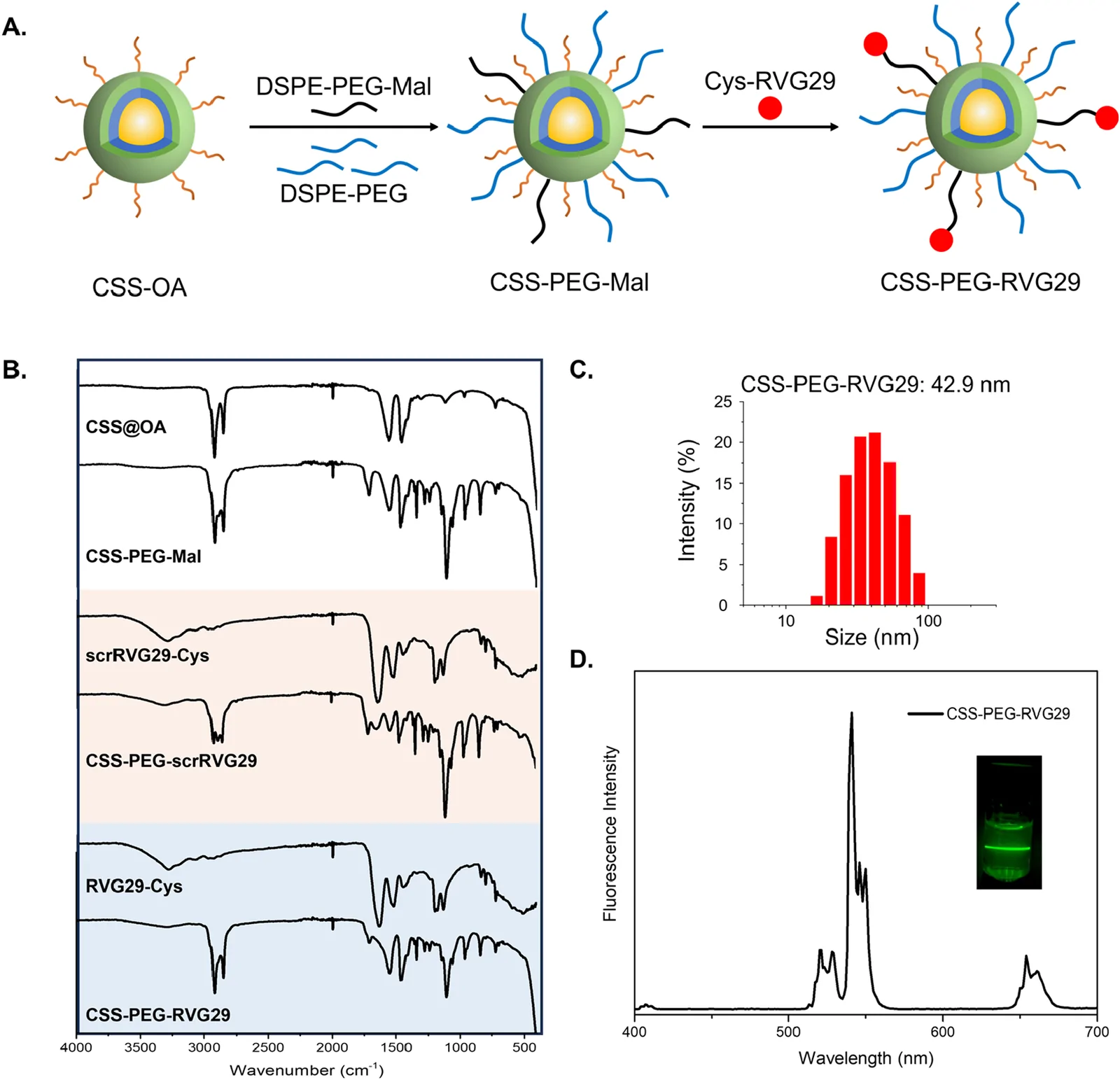
Synthesis route and characterization of CSS-PEG-RVG29: (A) Scheme of CSS-PEG-RVG29 synthesis; (B) FT-IR data show the conjugation of PEG-Mal, scrRVG29, and RVG29 with CSS nanoparticles; (C) graphics show the radius of UCNPs measured through DLS. (D) Upconversion spectra of CSS-PEG-RVG29 (1 mg/mL) were analyzed under 808 nm laser irradiation in aqueous solution (laser: 2.8 W/cm^2^); inset: photographs of CSS-PEG-RVG29 under an 808 nm laser (50 mW).

### 2.3 Neuronal Uptake of CSS-PEG-RVG29

To assess *in vitro* cytotoxicity, Neuro2a, NSC34, and C6 cells were incubated with the nanoparticles, followed by MTT assay analysis. The results showed that Neuro2a cells (Figure S3A), NSC34 cells (Figure S3B), and C6 cells (Figure S3C) were incubated with CSS-PEG-RVG29 at concentrations ranging from 0 to 500 μg/mL for 24 h. The findings indicated that cell viability remained above 80%, demonstrating the low cytotoxicity of the nanoparticles. Based on these findings, 100 μg/mL was selected as the working concentration of UCNPs for subsequent cellular uptake experiments.

Primary neurons, derived via direct isolation from fetal or neonatal CNS tissues, serve as a high-fidelity *in vitro* model for neurobiological research. By preserving the essential physiological and biochemical phenotypes of their *in vivo* counterparts, these cultures provide a robust platform for investigating neurodevelopmental trajectories and fundamental neuronal mechanisms. To establish high-fidelity *in vitro* models for evaluating UCNP internalization, primary neurons were seeded into 24-well plates at a density of 2 × 10^5^ cells per well. Between days 6 and 17 *in vitro*, both spinal cord (Figure S4) and cortical (Figure S6) populations exhibited extensive neurite outgrowth and the establishment of functional synaptic networks. Immunofluorescence staining verified that these neuronal populations were positive for Tuj1 (neuronal marker), as shown in Figures S5 and S7. Cells typically internalize particles through a process called endocytosis. A higher penetration and uptake of UCNPs are expected when RVG29 is present, as it facilitates endocytosis via nAChRs. Subsequently, cell uptake was tested, and confocal microscopy imaging revealed the uptake of CSS-PEG-RVG29 (100 μg/mL) in primary spinal (Figure 3A) and cortical neurons (Figure 3B) 24 h postincubation. In cortical neurons, the fluorescence intensity (FL) of CSS-PEG-Mal (FL: 9.9 ± 0.4 × 10^3^ a.u., *p* < 0.01) and CSS-PEG-scrRVG29 (FL: 10.0 ± 0.5 × 10^3^ a.u., *p* < 0.01) showed statistically significant increases relative to the vehicle (FL: 2.4 ± 0.3 × 10^3^ a.u.). In contrast, CSS-PEG-RVG29 (FL: 67.4 ± 5.9 × 10^3^ a.u., *p* < 0.01) elicited substantially greater uptake compared to the other three groups (Figure 3A,3C). In spinal neurons, CSS-PEG-RVG29 also produced a highly significant elevation in fluorescence intensity (FL: 79.7 ± 8.4 × 10^3^ a.u. *vs* 2.7 ± 0.1 × 10^3^ a.u. at vehicle, *p* < 0.01; *vs* 10.8 ± 0.4 × 10^3^ a.u. at CSS-PEG-Mal, *p* < 0.01; and *vs* 11.1 ± 0.6 × 10^3^ a.u. at CSS-PEG-scrRVG29, *p* < 0.01; Figure 3B,3D). These results demonstrate that the RVG29 peptide specifically enhances neuronal uptake of the CSS-PEG conjugate.

**Figure 3:**
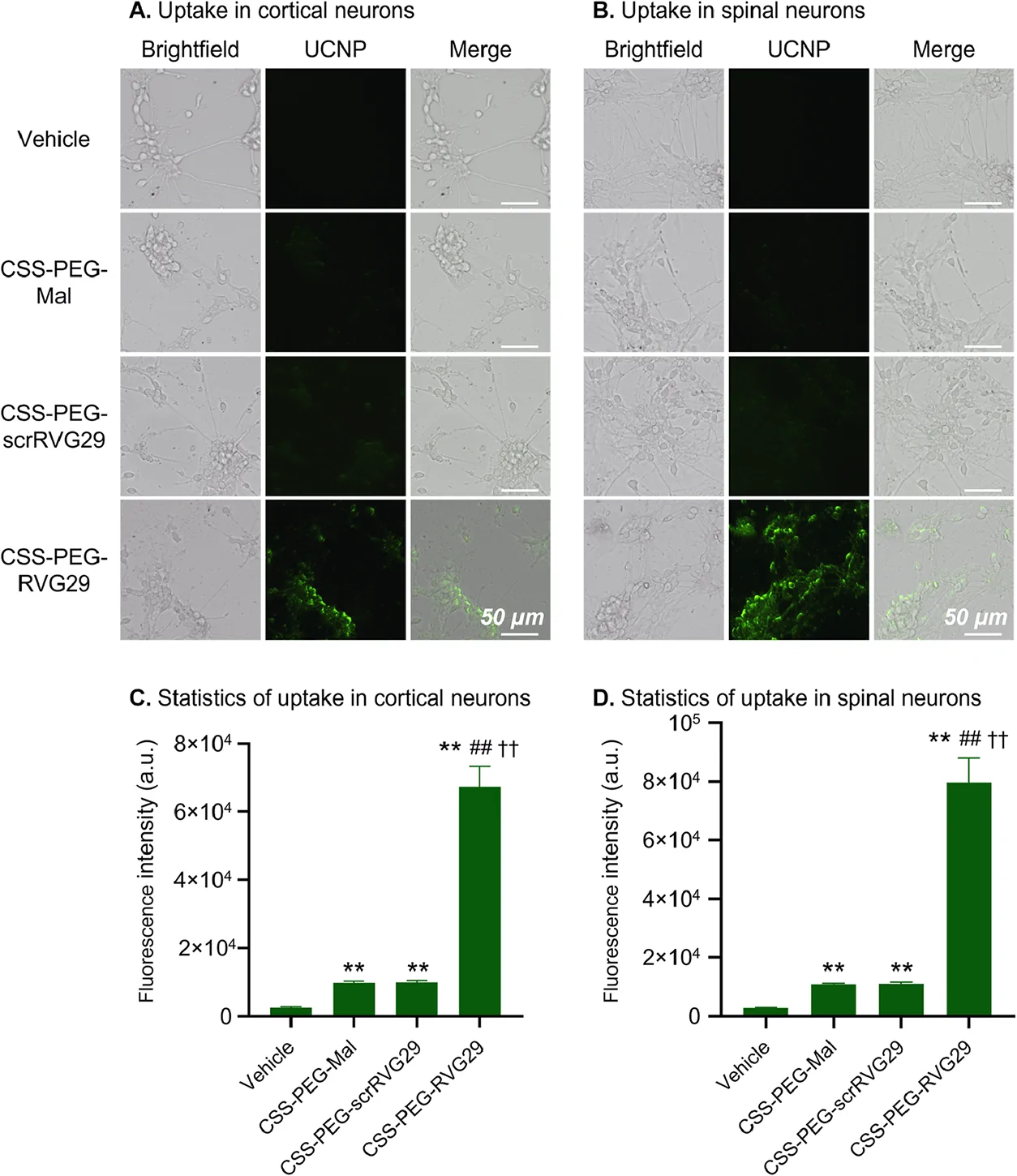
Uptake of CSS-PEG-Mal, CSS-PEG-scrRVG29, and CSS-PEG-RVG29 in (A) primary cortical neurons and (B) spinal neurons after 24 h of incubation. Green fluorescence images confirm UCNP uptake using multiphoton confocal microscopy. CSS-PEG-RVG29 exhibits markedly higher uptake by primary neurons than the other groups. Quantitative analysis of fluorescence intensity in (C) primary cortical neurons and (D) spinal neurons. Data are presented as mean ± SD (*n* = 5). Statistical analysis in (C, D) was performed using two-way ANOVA. **p < 0.01 *vs* vehicle; ^##^ *p* < 0.01 *vs* CSS-PEG-Mal; and ^††^ *p* < 0.01 *vs* CSS-PEG-scrRVG29.

### 2.4 Functional Assessment of Primary Neurons Exposed to CSS-PEG-RVG29

The whole-cell patch-clamp technique was employed to evaluate the impact of CSS-PEG-RVG29 on neuronal excitability and intrinsic firing properties. To assess the acute neurotoxicity and functional safety of the nanomaterials, primary spinal and cortical neurons were perfused with 100 μg/mL CSS-PEG-RVG29 in artificial cerebrospinal fluid (aCSF) for 10 min during recording. The extracellular aCSF solution consisted of 126 mM NaCl, 3 mM KCl, 1.3 mM MgSO_4_, 2.4 mM CaCl_2_, 1.3 mM NaH_2_PO_4_, 26 mM NaHCO_3_, and 10 mM glucose (pH 7.4; 305 mOsm/kg). Spontaneous action potentials were recorded using borosilicate glass electrodes (resistance 5–8 MΩ) filled with a standard intracellular solution to monitor real-time changes in membrane potential and discharge frequency. Our results revealed that CSS-PEG-RVG29 exposure significantly enhanced the firing rate of primary cortical neurons (1.27 ± 0.15-fold relative to baseline, *n* = 3, *p* = 0.0349 by a paired *t-*test; Figure 4A,4B). In contrast, no significant changes in firing rate or overall activity were observed in primary spinal neurons following CSS-PEG-RVG29 incubation (0.97 ± 0.06 relative to baseline, *n* = 3, *p* = 0.427 by a paired *t-*test; Figure 4C,4D). Collectively, these data indicate that RVG29 interactions with cortical neuronal receptors may modestly modulate neuronal excitability. The observed increase in action potential firing rate in cortical neurons, but not in spinal neurons, suggests that the effect of CSS-PEG-RVG29 is both specific and subtle, potentially due to differences in the expression of RVG29 target receptors or variations in downstream signaling pathways between these two types of neurons. This cell-type-specific response highlights the importance of neuronal heterogeneity in influencing the functional outcomes of interactions with nanomaterials. The 10 min postexposure interval was chosen to evaluate the acute, direct effects of CSS-PEG-RVG29 on neuronal membrane excitability, while minimizing any potential confounding influences of delayed transcriptional or translational changes. This time frame allows for the detection of rapid, receptor-mediated modifications in ion channel function following exposure to CSS-PEG-RVG29. In addition, previous studies have demonstrated that the engagement of cell-surface receptors by bioactive nanomaterials^36^ or ligands^37–39^ can induce electrophysiological changes within minutes of application. This further supports the relevance of the chosen time window. Together, our findings provide initial evidence for a modest, rapid, and cell-type-specific modulation of neuronal activity by CSS-PEG-RVG29, which justifies further investigation into the underlying mechanisms of this interaction.

**Figure 4:**
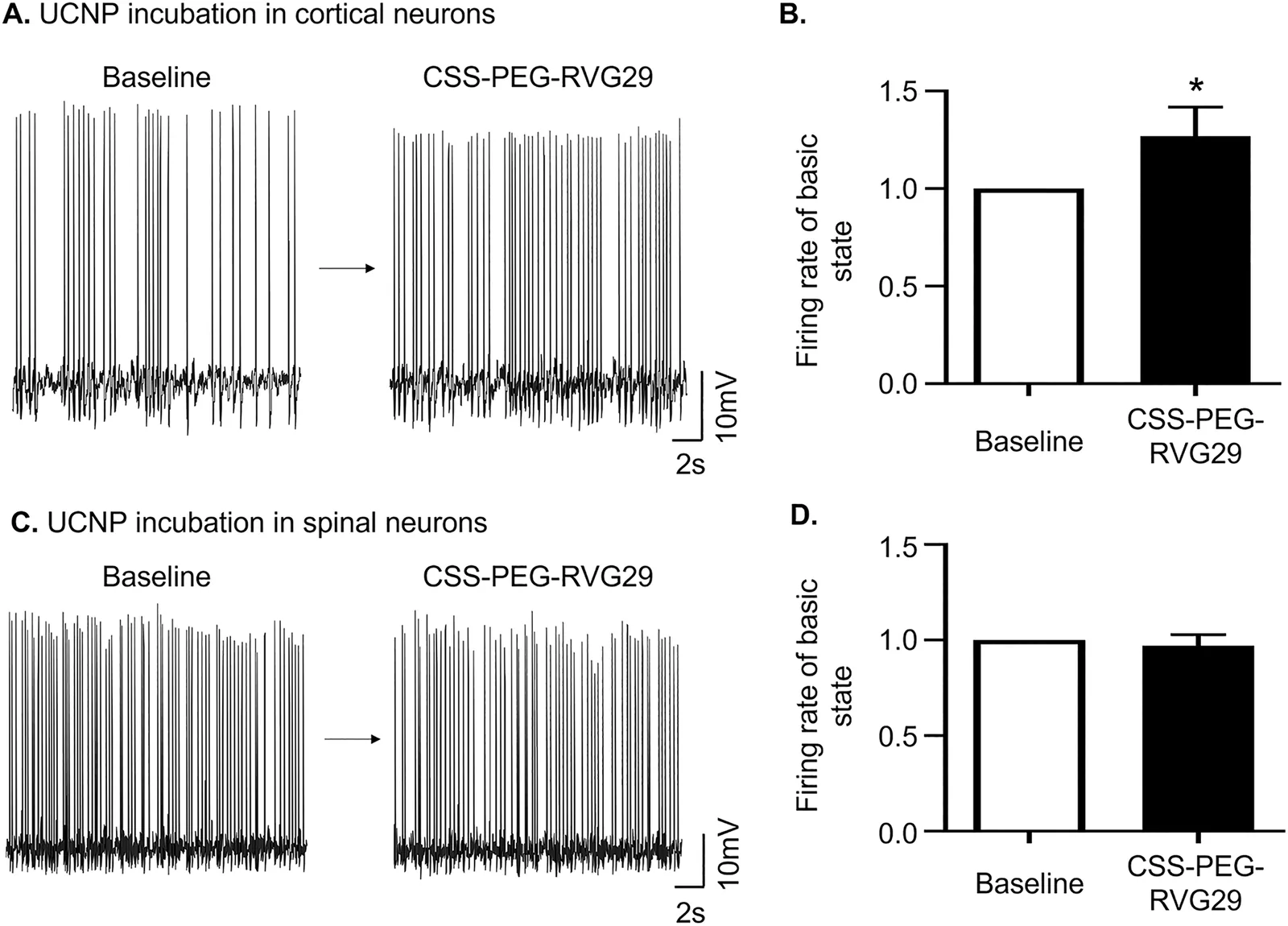
Action potential discharges in primary cortical and spinal neurons before and after incubation with CSS-PEG-RVG29. (A) Representative current-clamp recordings from a cortical neuron prior to and following exposure to 100 μg/mL CSS-PEG-RVG29. (B) Quantification of the relative firing rate of cortical neurons after 10 min of incubation of CSS-PEG-RVG29 (*n* = 3). (C) Representative current-clamp traces from a spinal neuron, recorded before and after incubation with 100 μg/mL CSS-PEG-RVG29. (D) Quantitative analysis of the relative firing rate in spinal neurons following 10 min of CSS-PEG-RVG29 exposure (*n* = 3). Statistical analysis in (B, D) was performed using a paired *t-*test. *p < 0.05.

### 2.5 Local Distribution of CSS-PEG-RVG29 Nanoparticles

*In vitro* results demonstrated that UCNPs conjugated with scrRVG29 exhibited no difference in neuronal uptake compared to those without RVG29 (Figure 3), suggesting that scrRVG29 lacks receptor-binding capability. To evaluate the local distribution of CSS-PEG-RVG29 and CSS-PEG-scrRVG29, we established four *in vivo* mouse experimental groups based on the treatment and time point: CSS-PEG-RVG29 (Day 1), CSS-PEG-RVG29 (Day 5), CSS-PEG-scrRVG29 (Day 1), and CSS-PEG-scrRVG29 (Day 5). Following intrafascicular injection into the right sciatic nerve (Figure 5A,5B), mice were euthanized on Day 1 or Day 5 postinjection. The right sciatic nerves were harvested to assess local distribution, while the noninjected contralateral left sciatic nerves served as internal controls. As shown in Figure 5C,5D, the injected (ipsilateral) sciatic nerve of mice treated with CSS-PEG-RVG29 displayed intense fluorescence on Day 1 (FL: 215.9 ± 40.0 × 10^3^ a.u.), which significantly decreased by Day 5 (FL: 29.1 ± 3.6 × 10^3^ a.u., *p* < 0.01). In contrast, mice receiving CSS-PEG-scrRVG29 exhibited markedly higher fluorescence intensity on Day 1 (FL: 597.0 ± 16.3 × 10^3^ a.u.) than the CSS-PEG-RVG29 group (*p* < 0.01). Although the fluorescence signal of the CSS-PEG-scrRVG29 group also declined significantly from Day 1 to Day 5 (FL: 179.8 ± 13.1 × 10^3^ a.u., *p* < 0.01), it remained substantially higher than that of the CSS-PEG-RVG29 group on Day 5 (*p* < 0.01). The contralateral sciatic nerves of CSS-PEG-RVG29 and CSS-PEG-scrRVG29 showed no detectable fluorescence at either time point (Figure S8). These data indicate that CSS-PEG-RVG29 is confined to the injected nerve within the first day and undergoes progressive retrograde transport to the spinal cord and higher CNS structures over time, but not to the contralateral site. CSS-PEG-scrRVG29 without functional RVG29 conjugation exhibits prolonged retention at the sciatic nerve.

**Figure 5:**
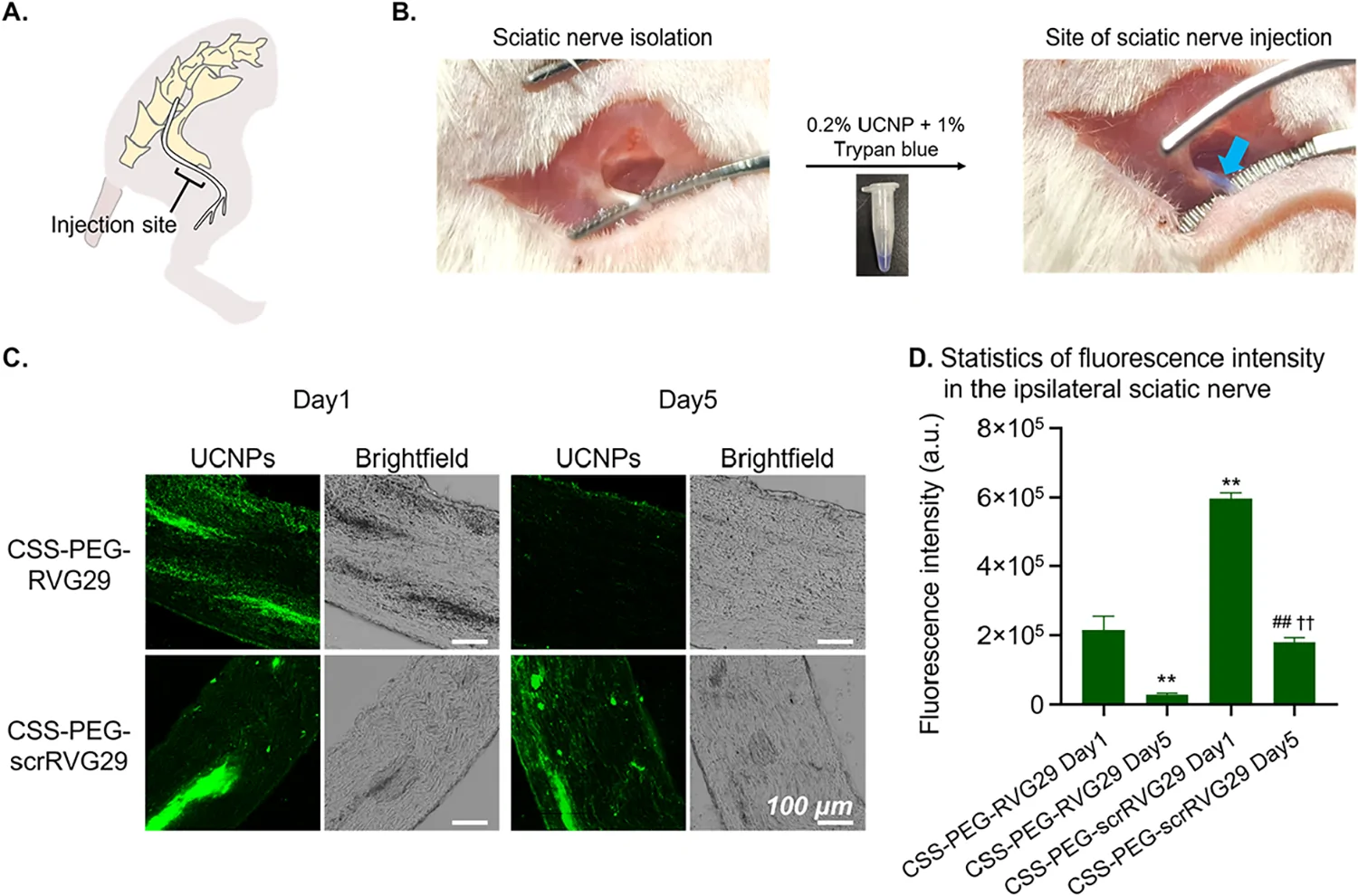
(A) Schematic figure of the sciatic nerve and injection site. (B) Right sciatic nerve exposed and injected with 2 mg/mL (0.2%) UCNPs and 1% Trypan blue (2 μL solution). (C) Confocal imaging of right (ipsilateral to the injection site) sciatic nerve tissue slices at Days 1 and 5 postinjection, injected with 2 μL of CSS-PEG-RVG29 or CSS-PEG-scrRVG29 solution (2 mg/mL). (D) Quantitative analysis of fluorescence intensity in the right sciatic nerve slices. Data are presented as mean ± SD (*n* = 5). Statistical analysis in (D) was performed using two-way ANOVA. **p < 0.01 *vs* CSS-PEG-RVG29 Day1; ^##^ *p* < 0.01 *vs* CSS-PEG-RVG29 Day5; ^††^ *p* < 0.01 *vs* CSS-PEG-scrRVG29 Day1.

### 2.6 *In Vivo* Neuronal Retrograde Transport of CSS-PEG-RVG29 Nanoparticles

The sciatic nerve conveys peripheral sensory information from the hindlimb to the spinal cord via the dorsal root ganglion and entry zone. These “primary afferents” ascend ipsilaterally within the gracile fasciculus of the dorsal column. Therefore, UCNPs’ biodistribution within the lumbar spinal cord was monitored at Days 1 and 5 postinjection (Figure 6A). The one-day time point was chosen to evaluate early retrograde axonal transport,^40^ while the five-day time point allowed assessment of continuous accumulation of nanoparticles by retrograde transport, subsequent trans-synaptic propagation, and longer-term distribution within the nervous system.^41^ UCNPs accumulation was assessed by fluorescence intensity. The *ex vivo* results revealed no significant fluorescence enhancement in the dorsal lumbar spinal cord on Day 1 postinjection after administration of either CSS-PEG-RVG29 (FL: 5.5 ± 0.5 × 10^3^ a.u.) or CSS-PEG-scrRVG29 (FL: 0.0 ± 0.0 × 10^3^ a.u.; Figure 6B,C). In contrast, by Day 5, the CSS-PEG-RVG29 group displayed markedly elevated fluorescence intensity (FL: 132.3 ± 8.6 × 10^3^ a.u.), which was significantly higher than both its own Day 1 value (*p* < 0.01) and that of the CSS-PEG-scrRVG29 group on Day 5 (FL: 5.7 ± 0.5 × 10^3^ a.u.; *p* < 0.01; Figure 6B,6C). These results indicate enhanced retrograde axonal transport and greater nanoparticle accumulation mediated by the RVG29 peptide.

**Figure 6:**
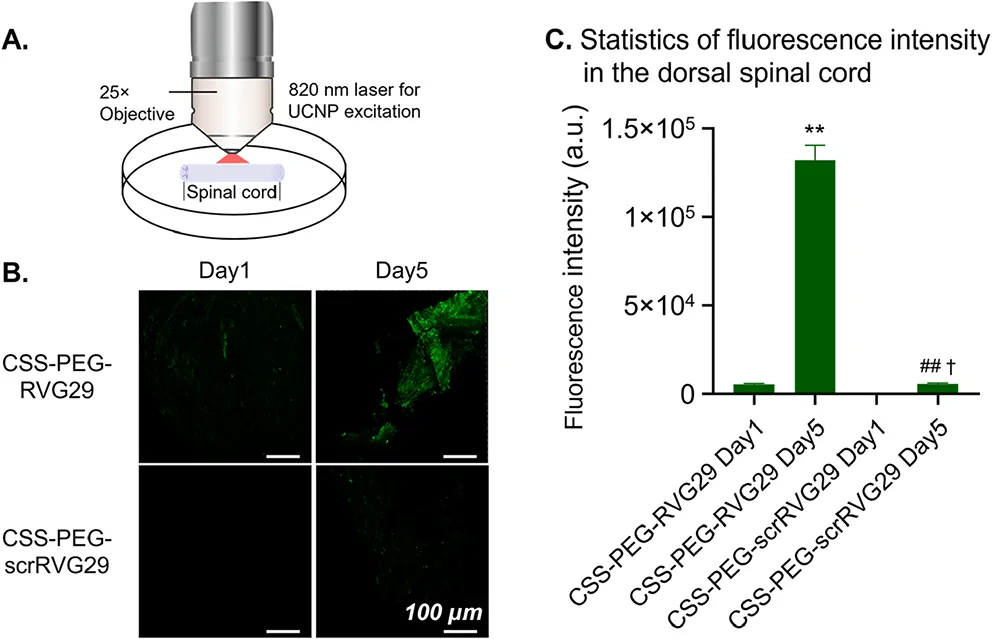
*Ex vivo* fluorescence imaging of the dorsal spinal cord. (A) Schematic of the two-photon imaging setup. (B) Representative 3D images of the dorsal lumbar spinal cord 1 or 5 days after the sciatic nerve injection of CSS-PEG-RVG29 or CSS-PEG-scrRVG29. Excitation wavelength: 820 nm, emission wavelength: 506–594 nm. Z-stack scanning thickness: 60 μm. (C) Quantitative analysis of fluorescence intensity in the dorsal spinal cord. Data are presented as mean ± SD (*n* = 3). Statistical analysis in (C) was performed using two-way ANOVA. **p < 0.01 *vs* CSS-PEG-RVG29 Day 1; ##*p* < 0.01 *vs* CSS-PEG-RVG29 Day 5; and ^†^ *p* < 0.05 *vs* CSS-PEG-scrRVG29 Day 1.

### 2.7 *In Vivo* Trans-synaptic Translocation of CSS-PEG-RVG29 Nanoparticles

The nerves of the gracile fasciculus form synapses onto second-order neurons in the gracile nucleus of the caudal medulla. The decussated axons of these “second-order neurons” then ascend via the medial lemniscus to terminate in the ventral posterolateral (VPL) nucleus of the thalamus. From there, “third-order neurons” project to the hindlimb representation within the primary somatosensory cortex (S1HL).^42,43^ For the *ex vivo* studies, mice were deeply anesthetized and euthanized, and their brains were harvested. Subsequently, the brains were dehydrated in 30% sucrose solution and sectioned according to “The Mouse Brain in Stereotaxic Coordinates, Second Edition”^44^ for imaging of the thalamus VPL and S1HL. At Day 5 postinjection, CSS-PEG-RVG29 was detected in the left VPL (FL: 2489.3 ± 174.5 a.u.) and left S1HL (FL: 245.3 ± 20.1 a.u.) regions of the mouse brain (Figure 7A,7C). Fluorescence intensity in the left VPL was significantly higher than that in the right VPL (FL: 41.3 ± 6.1 a.u., *p* < 0.05) and in the left VPL of the CSS-PEG-scrRVG29 group (FL: 49.3 ± 8.3 a.u., *p* < 0.05). Likewise, intensity in the left S1HL was significantly higher than that in the right S1HL (FL: 50.7 ± 6.1 a.u., *p* < 0.05) and in the left S1HL of the CSS-PEG-scrRVG29 group (FL: 42.7 ± 6.3 a.u., *p* < 0.05; Figure 7A–C). These findings demonstrate that RVG29 facilitates retrograde transport and trans-synaptic delivery of nanoparticles within neuronal pathways and networks. At Day 5 postinjection, fluorescence intensity in the left S1HL was lower than in the left VPL (*p* < 0.05). This pattern aligns with the somatosensory relay from the sciatic nerve: primary afferents first synapse in a spinal/medullary relay, second-order neurons then synapse in VPL (two synapses from the periphery), and third-order thalamocortical neurons project to S1HL (three synapses from the periphery). Because S1HL is one synapse downstream of VPL, nanoparticles must undergo an additional trans-synaptic translocation, leading to further dilution and dispersion across broader thalamocortical projections, thereby lowering cortical fluorescence intensity.

**Figure 7:**
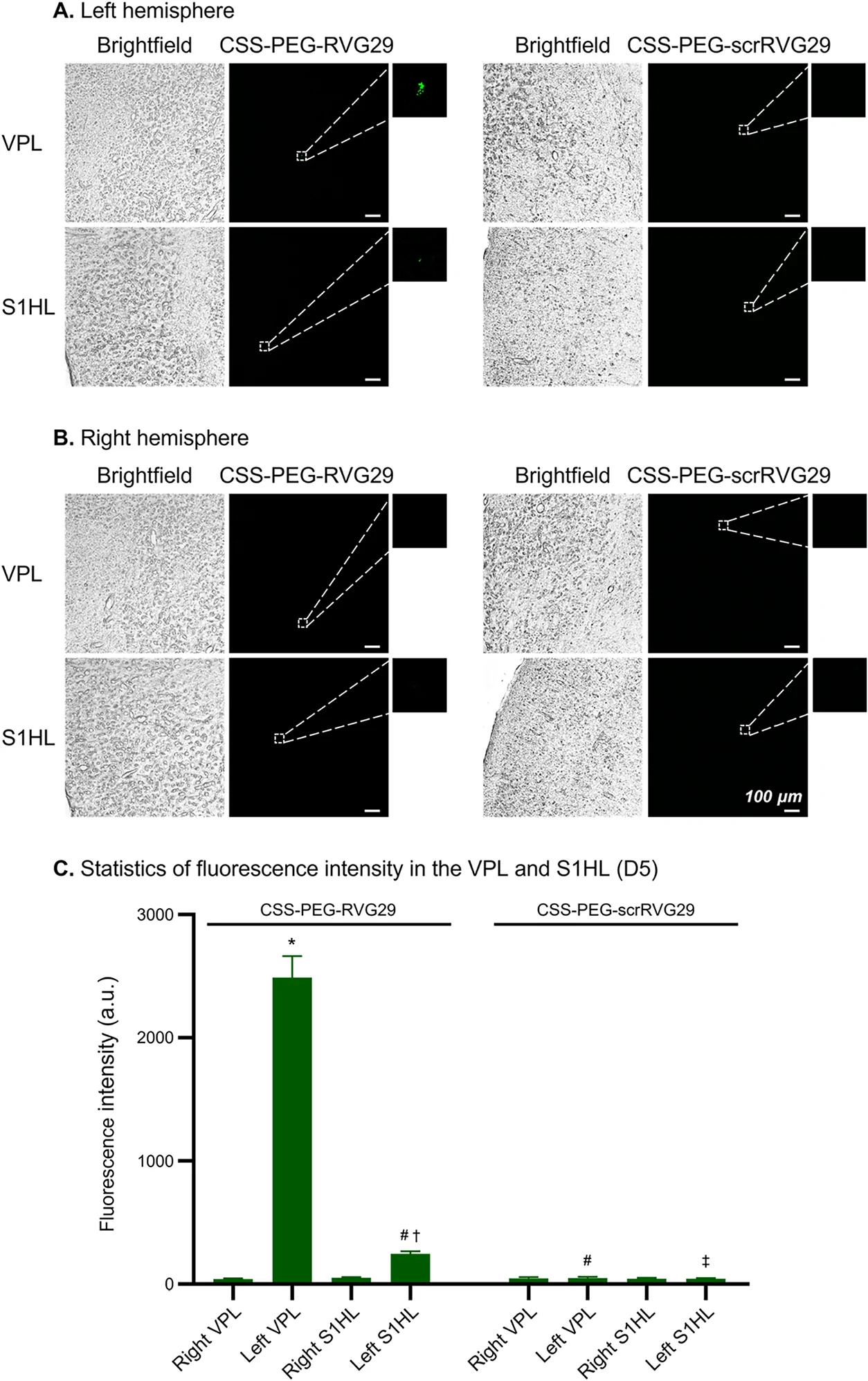
Multiphoton confocal images of the thalamic ventral posterolateral nucleus (VPL) and somatosensory cortex (S1HL) in mouse brain sections, 5 days after intrafascicular sciatic nerve injection. (A) Contralateral (left) to the injected nerve. (B) Ipsilateral (right). (C) Quantitative analysis of fluorescence intensity in the VPL and S1HL. Data are presented as mean ± SD (*n* = 3). Statistical analysis in (C) was performed using two-way ANOVA. *p < 0.05 *vs* right VPL, ^#^ *p* < 0.05 *vs* left VPL, ^†^ *p* < 0.05 *vs* right S1HL, and ^‡^ *p* < 0.05 *vs* left S1HL of CSS-PEG-RVG29. Accumulation of CSS-PEG-RVG29 was observed in the left VPL and S1HL regions, whereas no accumulation of CSS-PEG-scrRVG29 was detected in these regions. This finding indicates effective retrograde and trans-synaptic translocation of RVG29-conjugated UCNPs.

#### 2.7.1 Precise Neural Circuit Delivery with Negligible Systemic Exposure

To evaluate potential systemic exposure after intrafascicular injection, we measured organ-level fluorescence at days 1 and 5 postinjection. No detectable accumulation of CSS-PEG-RVG29 was observed in the liver, spleen, or kidney (Figure S9), indicating no measurable entry into the circulation and thus no evidence of systemic exposure within the sensitivity of our assay.

### 2.8 Discussion

Synthetic UCNPs are typically capped with long-chain hydrophobic ligands, which limit their solubility to nonpolar solvents and require surface modification for their biological utility. Such modifications are critical not only for ensuring dispersibility in aqueous media but also for mitigating the risk of nonspecific interactions between unprotected surfaces and intracellular biomolecules, which can trigger cytotoxicity.^45^ Our results align with previous findings that functionalization with biocompatible polymers, such as PEG,^46^ effectively enhances water dispersibility while significantly reducing toxicological impact. Furthermore, the integration of targeting ligands, including peptides, underscores the versatility of UCNPs for advanced preclinical imaging applications.^33,34^

In this study, we synthesized CSS structure UCNPs (NaYF_4_:YbEr@NaYF_4_:YbNd@NaYF_4_) via high-temperature thermal decomposition, achieving several complementary advantages. Sequential shell growth achieved upconversion luminescence efficiency under 808 nm laser excitation, generating strong green emission (500–575 nm) optimal for deep-tissue multiphoton microscopy, with minimal autofluorescence and phototoxicity. The outer NaYF_4_ shell increases photostability and suppresses surface quenching. Surface functionalization was achieved by encapsulation with the amphiphilic ligand DSPE-PEG, which introduced PEG to enhance colloidal stability and provided terminal maleimide groups for RVG29 peptide conjugation, yielding CSS-PEG-RVG29. This modification not only improved the biocompatibility of UCNPs but also enabled neuron-specific targeting of nAChRs. Functionalized UCNPs could be employed as dual-purpose agents, acting as both fluorescent tracers and model carriers to validate the platform’s cargo delivery efficacy. CSS-PEG-RVG29 displayed several advantageous properties, including a small hydrodynamic radius (42.9 nm) and low cytotoxicity. *In vitro* assays demonstrated minimal toxicity to both neuronal (Neuro2a, NSC34) and glial (C6) cell lines at concentrations up to 500 μg/mL. Whole-cell patch-clamp recordings further confirmed that CSS-PEG-RVG29 did not alter the electrophysiological properties of spinal neurons, supporting its biocompatibility. These findings address common concerns about the neurotoxicity of nanomaterials, including oxidative stress and ion channel disruption, and highlight the translational potential of CSS-PEG-RVG29 for neurotherapeutic applications.

We systematically evaluated the neuronal uptake of the constructed UCNPs using primary neurons derived from the mouse spinal cord and cortex. Enhanced cellular uptake of CSS-PEG-RVG29 was observed in all tested neuronal cell types, reflecting the neuron-specificity afforded by RVG29, which could mimic the virus’s neuronal tropism via a molecular Trojan horse mechanism, greatly enhancing neuronal affinity and delivery efficiency.^47,48^ RVG29 facilitates neuron-specific targeting by interacting with cell-surface nAChRs, particularly the α7 subtype, to trigger receptor-mediated endocytosis. The internalization of RVG29-conjugated nanoparticles is an active, energy-dependent process that utilizes distinct endocytic pathways, including macropinocytosis, micropinocytosis, lipid-raft-, caveolae-, clathrin-, and acid-sensing ion channel-mediated endocytosis, contingent upon the physicochemical properties of the nanoparticles and the target cell phenotype.^49–51^

Furthermore, we demonstrated that CSS-PEG-RVG29 is capable of axonal retrograde transport and trans-synaptic translocation *in vivo*. Following sciatic nerve injection in mice and subsequent two-photon imaging, nanoparticles were clearly detected within the dorsal spinal cord and accumulated in higher order brain regions, including the thalamus VPL and the somatosensory cortex (S1HL). This indicates that the platform effectively enables delivery in peripheral and central neural pathways. Mechanistically, the system initiates clathrin-mediated endocytosis at axon terminals, followed by endosomal escape and dynein-mediated retrograde transport along microtubules.^20^ Subsequent synaptic transfer enables spreading to secondary and tertiary neurons, imitating the neurotropic behavior of the rabies virus but without viral cytotoxicity.

In our previously published *in vitro* synapse model (Liu et al.), RVG29-conjugated polymers exhibited progressive accumulation within presynaptic neurons from day 1.5 to day 7;^52^ this accumulation was substantially abolished by scrRVG29, thereby confirming the RVG29-dependent crossing of the synaptic cleft. Consistent with these findings, the sequential appearance of nanoparticles along predefined neural pathways, detected by fluorescence imaging, provides clear evidence of trans-synaptic transport.^53,54^ In addition, following entry into the brain parenchyma, inorganic nanoparticles such as UCNPs are primarily cleared via the paravascular glymphatic pathway. After microglial internalization, the particles are shuttled toward perivascular spaces and subsequently drain into meningeal lymphatic vessels and deep cervical lymph nodes.^55^

Moreover, the nanoparticles showed no accumulation in the spleen, liver, or kidneys. Compared to systemic or intrathecal administration, intraneural injection is minimally invasive, exploiting the native architecture of peripheral nerves for delivery and reducing off-target exposure. However, clinically relevant cargoes with differing physicochemical properties may exhibit distinct transport efficiencies and peripheral accumulation profiles;^56,57^ nevertheless, intraneural injection enables more precise targeting and effectively minimizes off-target peripheral exposure.

### 2.9 Conclusions

In conclusion, our developed CSS-PEG-RVG29 platform bridges the gap between peripheral administration and CNS delivery. These functionalized UCNPs serve as a bifunctional platform for real-time monitoring and model cargo transport, demonstrating the system’s versatility for therapeutic delivery. The combination of minimal cytotoxicity and preserved electrophysiological function underscores its safety, while its ability to navigate trans-synaptic pathways highlights its functional precision. By enabling efficient transport to the spinal cord and brain from the periphery, this architecture offers significant promise for overcoming the blood–brain barrier in future clinical and diagnostic neuroapplications.

### 2.10 Materials and Methods

All animal experiments followed Neuroscience Research guidelines and were approved by the Institutional Animal Care and Use Committees at the Hong Kong Ministry of Health and Hong Kong Baptist University (Protocol: (23–139) in DH/HT&A/8/2/6 Pt.8).

#### 2.10.1 Synthesis of CSS-PEG-Mal

To enhance biocompatibility, the CSS nanoparticles were encapsulated with the amphiphilic ligand DSPE-PEG. A mixture was prepared by combining 10 mg of CSS, 5 mg of DSPE-PEG, and 5 mg of DSPE-PEG-Mal (maleimide, Mal) in 10 mL of chloroform, which was stirred at room temperature overnight. Once the organic solvent has evaporated and dried, 10 mL of deionized water was added. The mixture was then centrifuged at 8000 rpm for 10 min to remove any unencapsulated ligands. This process is repeated three times, after which CSS-PEG-Mal was dispersed in the DMEM for storage.

#### 2.10.2 Synthesis of CSS-PEG-RVG29 and CSS-PEG-scrRVG29

The RVG29 peptide (YTIWMPENPRPGTPCDIFTNSRGKRASNG) or scrRVG29 (WTMGPYNRPPGTDCSIFNRGTKRASNG) with a C-terminal cysteine was conjugated with CSS-PEG-Mal via thiol-maleimide chemistry. 5 mg of CSS-PEG-Mal in phosphate-buffered saline (pH 7.4) was reacted with 5 mg of peptide at room temperature for 24 h in the dark. Unreacted peptide was removed by centrifuging. Conjugation efficiency was assessed by FT-IR for both CSS-PEG-RVG29 and CSS-PEG-scrRVG29.

#### 2.10.3 Characterization of UCNPs

##### 2.10.3.1 Structural and Morphological Characterization

The crystalline phase of the synthesized UCNPs was determined via X-ray diffraction (XRD) by using a Bruker D8 Focus diffractometer (Cu K radiation). Morphological analysis and particle sizing were performed by using a Tecnai G2 S-TWIN transmission electron microscope (TEM) operated at 200 kV, supplemented by dynamic light scattering (DLS) measurements on a DelsaMax Core analyzer. Surface functional groups were identified using a PerkinElmer Spectrum Two Fourier transform infrared (FT-IR) spectrometer.

##### 2.10.3.2 Spectroscopy

Upconversion emission spectra were acquired by using a Horiba FluoroMax-4 photoluminescence spectrometer. Upconversion luminescence was specifically induced by using an external 808 nm continuous-wave laser diode (Blueprint, China).

#### 2.10.4 Cell Uptake

Primary spinal and cortical neurons were incubated separately with 100 μg/mL CSS-PEG-Mal, CSS-PEG-scrRVG29, or CSS-PEG-RVG29 for 24 h. Following incubation, the culture medium was aspirated, and the cells were washed with phosphate-buffered saline. Cellular uptake of the UCNPs was evaluated utilizing a Leica TCS SP8MP multiphoton/confocal microscope (Leica Microsystems, Wetzlar, Germany) equipped with a 20× objective.

#### 2.10.5 Patch Clamp

Electrophysiological characterization was performed via patch-clamp recordings, utilizing high-resistance giga-ohm seals between glass micropipettes and the plasma membrane. This approach enabled the high-resolution monitoring of transmembrane ionic flux and action potential kinetics, providing quantitative insights into ion channel conductance and intrinsic neuronal excitability. For *in vitro* electrophysiological assessments, 100 μg/mL CSS-PEG-RVG29 was introduced into the aCSF perfusion solution, and primary spinal and cortical neurons were exposed to this solution for 10 min during recording. All recordings were compensated for liquid junction potential. Data acquisition and analysis were performed using Clampex 10 and Clampfit 700B software.

#### 2.10.6 Surgical Exposure and Intrafascicular Injection of the Sciatic Nerve

CD-1 mice were deeply anesthetized with an intraperitoneal injection of ketamine (75 mg/kg), xylazine (8.3 mg/kg), and acepromazine (1.6 mg/kg). While prone, a linear incision was made from the ilium to the ischium, and the right sciatic nerve was exposed by bluntly dissecting between the gluteus superficialis and biceps femoris muscles, followed by partial displacement of the gluteus superficialis and separation of the gluteus medius and piriformis. The sciatic nerve was visualized from the proximal segment under the gluteus medius to the distal segment above the obturator and gemelli muscles and then gently freed from the fascia for injection.

Mice were randomly assigned to four groups:^58^ (1) CSS-PEG-scrRVG29 (2 μL, 2 mg/mL) at Day 1 postinjection, (2) CSS-PEG-scrRVG29 (2 μL, 2 mg/mL) at Day 5 postinjection, (3) CSS-PEG-RVG29 (2 μL, 2 mg/mL) at Day 1 postinjection, and (4) CSS-PEG-RVG29 (2 μL, 2 mg/mL) at Day 5 postinjection. Intrafascicular injections were performed into the right sciatic nerve, 5–10 mm proximal to the obturator fascia. After injection, the muscles were repositioned and sutured, and the skin was then closed with sutures and thoroughly disinfected.

#### 2.10.7 Multiphoton Microscopy Imaging

Brain and sciatic nerve sections were imaged on a multiphoton laser-scanning microscope with 808 nm excitation and emission collected at 500–575 nm. The lumbar spinal cord was imaged with 820 nm excitation, and emission was collected at 506–594 nm. Z-stack image series were acquired to reconstruct the 3D distribution of the UCNPs within the spinal cord.

#### 2.10.8 Statistical Analysis

For MTT, patch-clamp, and multiphoton imaging experiments, data are presented as mean ± standard deviation (SD), and all analyses were conducted using GraphPad Prism 8 software. Specifically, in the MTT assay, raw absorbance values were first blank-corrected by subtracting the mean absorbance of blank wells. The blank-corrected values were then normalized to the mean absorbance of the vehicle control wells on the same plate (set to 100%) to minimize interplate variation. In patch-clamp recordings, neuronal firing rates were measured and normalized to the baseline firing rate (set as 1.0).^59^ Comparisons between values obtained before and after UCNPs incubation in the patch clamp were performed using a paired *t-*test. In the multiphoton imaging experiment, fluorescence intensity was quantified using ImageJ software by measuring pixel values. For primary neurons, sciatic nerve, and dorsal spinal cord sections, regions of interest comprising 7225 pixels were analyzed. For brain sections, regions of interest comprising 400 pixels in the vicinity of the fluorescent signal were selected for the measurement. Statistical analyses in multiphoton imaging were performed using two-way analysis of variance (ANOVA) to compare differences among multiple groups. When the assumption of homogeneity of variance was satisfied, Fisher’s least significant difference (LSD) post hoc test was applied for pairwise comparisons. When variances were unequal, Tamhane’s T2 test was used instead. *p < 0.05* was considered statistically significant.

## Supplementary Material



## Data Availability

The data that support the findings of this study are available from the corresponding author upon reasonable request.

## Notes

Experimental methods detailing materials, cell culture, cytotoxicity assays, isolation and culture of primary mouse neurons, immunocytochemistry, sample collection and processing, and multiphoton imaging (PDF)
