# Supplementary material for: Transneuronal Transport of Ligand-Conjugated Upconversion Nanoparticles from the Peripheral to the Central Nervous System
Source: JACS Au. 2026 Aug 20;6(9):5295–306. doi: 10.1021/jacsau.6c00949 (PMC13625534; doi:10.1021/jacsau.6c00949)
Supplement: Supplementary file 1 [file au6c00949_si_001.pdf]

Supplementary Information

Transneuronal Transport of Ligand-Conjugated Upconversion Nanoparticles from the Peripheral to the Central Nervous System

Haitao Liu,[1#] Hengde Li,[1#] Xi Chen,[1] Saman Hamidi,[2] and Angelo H. ALL[1*]

1 Department of Chemistry, Hong Kong Baptist University, Kowloon, Hong Kong SAR 999077, China

2 Morsani College of Medicine, University of South Florida, Tampa, Florida 33602, United States

# Contributed equally and share the first authorship

** Corresponding Authors: Angelo H. ALL*

(angelo@hkbu.edu.hk - ORCID: 0000-0002-7086-2170)

Angelo H. ALL, MD, MBA
E-mail: angelo@hkbu.edu.hk
ORCID: 0000-0002-7086-2170
Institutional title: Associate Professor
Affiliation: Department of Chemistry, Hong Kong Baptist University, Hong Kong
Address: Sir Run Run Shaw Building, Office RRS844, Ho Sin Hang Campus, Hong Kong Baptist University, 224 Waterloo Road, Kowloon, Hong Kong SAR 999077, China
Direct office: (+852) 34115956

Haitao Liu
E-mail: 22482571@life.hkbu.edu.hk
Institutional title: PhD Candidate
Affiliation: Department of Chemistry, Hong Kong Baptist University, Hong Kong
Address: Cha Chi-ming Science Tower, Office SCT805, Ho Sin Hang Campus, Hong Kong Baptist University, 224 Waterloo Road, Kowloon, Hong Kong SAR 999077, China

Hengde Li
E-mail: 23483652@life.hkbu.edu.hk
Institutional title: PhD Candidate
Affiliation: Department of Chemistry, Hong Kong Baptist University, Hong Kong
Address: Cha Chi-ming Science Tower, Office SCT805, Ho Sin Hang Campus, Hong Kong Baptist University, 224 Waterloo Road, Kowloon, Hong Kong SAR 999077, China

Xi Chen
E-mail: 25482831@life.hkbu.edu.hk
Institutional title: PhD student
Affiliation: Department of Chemistry, Hong Kong Baptist University, Hong Kong
Address: Cha Chi-ming Science Tower, Office SCT805, Ho Sin Hang Campus, Hong Kong Baptist University, 224 Waterloo Road, Kowloon, Hong Kong SAR 999077, China

Saman Hamidi
E-mail: samanhamidi@usf.edu
Institutional title: MD student
Affiliation: Morsani College of Medicine, University of South Florida
Address: 560 Channelside Drive, Tampa, FL 33602, United States

Supplementary Methods

Materials and Instruments

Materials

All solvents and chemicals were purchased from commercial suppliers in analytical grade and used without further purification unless special stated. DSPE-PEG2000 and DSPE-PEG2000-Mal were purchased from Xi'an Ruixi Biological Technology Co.,Ltd. RVG29-Cys and scrambled RVG29-Cys (Sequence: WESYRTRAIPKCSPGTDPMINPFTRGNGNC) were purchased from ChinaPeptides Co., Ltd. Dulbecco's Modified Eagle's Medium (DMEM), trypsin-EDTA (0.25%), Dulbecco's phosphate-buffered saline (PBS), penicillin-streptomycin, fetal bovine serum (FBS), Neurobasal Plus medium, and Triton X-100 were purchased from Thermo Fisher Scientific Inc. DAPI (4',6-diamidino-2-phenylindole). Neuronal class III β3-Tubulin (Tuj1) mouse monoclonal antibody (Cat#AT809) was from Beyotime. Urethane and bovine serum albumin were purchased from Energy Chemical Co.,Ltd. Neuro-2a was from ATCC (CCL-131). Mouse neuron-like hybrid cell line (NSC-34) was from Cedarlane, Burlington, Canada (CLU140). C6 cells were from Fenghui Biotechnology Co., Ltd.

Synthesis of UCNPs

Synthesis of $NaYF_4$:YbEr core (C) nanoparticles.

A solution of lanthanide acetate (1 mmol), comprising 78% Y^{3+}, 20% Yb^{3+}, and 2% Er^{3+}, was prepared in water and combined with 6 mL of oleic acid (OA) and 15 mL of 1-octadecene (ODE) in a 100 mL two-neck flask. The mixture was heated to 150 °C under a nitrogen atmosphere for 60 min to remove water, resulting in a clear solution. After cooling to room temperature, 100 mg of NaOH and 148 mg of NH_4F in 6 mL of

methanol were incorporated. The resulting mixture was stirred at 50 °C for 30 min, then heated to 110 °C for 10 min. Following a 10-min degassing step, the reaction was further heated to 290 °C under nitrogen protection for 90 min. Once the reaction was complete and the mixture cooled, 20 mL of ethanol was added. The core UCNPs were isolated by centrifugation at 6000 rpm for 10 min after ultrasonic mixing. The UCNPs were then purified through redispersion in cyclohexane, followed by three times consecutive precipitation steps with ethanol, and finally dispersed in 4 mL of cyclohexane for storage.

***Synthesis of $NaYF_4$:YbEr@$NaYF_4$:YbNd core-shell (CS) nanoparticles*.**

A mixture containing 0.35 mmol of Y^{3+}, 0.05 mmol of Yb^{3+}, and 0.1 mmol of Nd^{3+} was prepared in a 100 mL two-neck flask by combining it with 3 mL of OA and 7 mL of ODE. The mixture was heated to 150 °C under a nitrogen flow to eliminate water, maintaining this temperature for 60 min. After cooling to 90 °C, 2 mL of core UCNPs suspended in cyclohexane was introduced to facilitate the removal of cyclohexane. Following a subsequent cooling to room temperature, 50 mg of NaOH and 74 mg of NH_4F dissolved in 3 mL of MeOH were added to the reaction mixture. The solution was stirred at 50 °C for 30 min, then heated to 110 °C for 10 min. After a degassing step of 10 min, the mixture was further heated to 290 °C for 90 min in a nitrogen atmosphere. Upon cooling to room temperature, 20 mL of ethanol was added, and the core-shell UCNPs were isolated *via* centrifugation at 6000 rpm for 10 min following ultrasonic mixing. Purification of the UCNPs was achieved by redispersing them in cyclohexane and precipitating with ethanol three times. The core-shell UCNPs were then dispersed in cyclohexane for storage.

Synthesis of $NaYF_4$:YbEr@$NaYF_4$:YbNd@$NaYF_4$ core-shell-shell (CSS) nanoparticles.

A solution containing 0.25 mmol of Y^{3+} in 3 mL OA and 7 mL ODE. The mixture

was heated to 150 °C for 60 min under a nitrogen atmosphere. After cooling to 90 °C, 100 mg of core-shell UCNPs suspended in cyclohexane was added. Following a subsequent cooling to room temperature, 50 mg NaOH and 74 mg NH_4F dissolved in 3 mL MeOH were introduced. The solution was stirred at 50 °C for 30 min before being heated to 110 °C for an additional 10 min. A degassing step of 10 min was then performed, after which the mixture was heated to 290 °C for 90 min in an inert nitrogen atmosphere. Upon cooling to room temperature, 20 mL of ethanol was added, and the core-shell-shell UCNPs were isolated by centrifugation at 6000 rpm for 10 min. The final core-shell-shell UCNPs were stored in cyclohexane for future use.

Cell Culture

Neuro2a, NSC34, and C6 cell lines were cultured in DMEM supplemented with 10% heat-inactivated FBS and 1% streptomycin/penicillin (10,000 U/mL). Cells were cultivated at 37 °C with 5% CO_2.

Cytotoxicity Assays

Cell viability was assessed *via* the MTT reduction assay. In brief, Neuro2a, NSC34, and C6 cells were seeded into 96-well plates at a density of 8×10^3 cells per well. Following a 24-h incubation, cells were exposed to varying concentrations of CSS-PEG-RVG29 and further incubated for an additional 24 h. Subsequently, 20 μL of MTT solution was introduced into each well, yielding a final concentration of 0.5 mg/mL. The plates were then incubated for 4 h at 37 °C in a humidified atmosphere containing 5% CO_2. After incubation, the medium was carefully removed, and 150 μL of DMSO was added to solubilize the formazan crystals. The plates were agitated for 10 min, after which absorbance was recorded at 492 nm using a microplate reader. Cell viability was

determined by normalizing the absorbance of treated samples to that of the control group. All data are expressed as mean ± standard deviation (SD) from four independent experiments.

Isolation and Culture of Primary Mouse Neurons

Primary cortical and spinal neurons were isolated from embryonic day 15 CD-1 mouse embryos. Brains and spinal cords were harvested and transferred to ice-cold DMEM. Cortical tissue was carefully dissected and subjected to enzymatic dissociation with 2 mg/mL papain and 125 U/mL DNase I in DMEM at 37 ℃ for 30 min, after which DMEM containing 10% FBS was added to terminate digestion. Spinal cords were processed similarly, with the addition of 0.125% collagenase IV during the enzymatic treatment. Cell suspensions were centrifuged at 1000 rpm for 5 min and resuspended in DMEM supplemented with 10% FBS and 125 U/mL DNase I. Cells were then counted and plated at 4×10^5 cells/mL, maintained at 37 °C in a humidified 5% CO_2 incubator. The following day, the medium was replaced with complete Neurobasal Plus medium (supplemented with 5% horse serum, 2% B27, 1% GlutaMAX, and 1% penicillin/streptomycin). Cultures were maintained in 24-well plates for 10 days to allow axon and neurite outgrowth before subsequent experiments.

Immunocytochemistry

Cells were washed with PBS and subsequently fixed with 4% paraformaldehyde in 0.1 M PBS for 15 min at room temperature. Following fixation, cells were rinsed again with PBS and permeabilized for 10 min using 0.1% Triton X-100 in 0.1 M PBS. After additional PBS washes, non-specific binding sites were blocked by incubating the cells with 1% bovine serum albumin in 0.1 M PBS for 2 h at room temperature. Cells were

then incubated with mouse anti-Tuj1 primary antibody (1:1000 dilution) overnight at 4°C. After three washes with PBS (5 minutes each), cells were exposed to goat anti-mouse secondary antibody conjugated to Alexa Fluor 488 (1:1000 dilution) for 1 hour at room temperature. Subsequently, cells were washed three times with PBS before nuclear staining with DAPI (1 μg/mL in 0.1 M PBS) for 10 minutes. Fluorescent images were acquired using a confocal microscope.

Sample Collection and Processing

Tissue samples, including the sciatic nerve, lumbar spinal cord, brain, spleen, liver, and kidney, were collected at Days 1 or 5 post-injection. After transcardial perfusion with paraformaldehyde (PFA), organs were carefully dissected and post-fixed in 4% PFA at 4 °C overnight. Subsequently, they were cryoprotected in 30% sucrose in PBS at 4 °C until fully saturated. Serial sections with a thickness of 20 μm were prepared using a cryostat and mounted onto glass slides for subsequent imaging analyses.

Multiphoton Imaging

Imaging of brain, sciatic nerve, spleen, liver, and kidney sections was conducted using a Leica TCS SP8 MP multiphoton/confocal microscope. Samples were excited at 808 nm, and fluorescence emission was detected within the 500-575 nm range. For *ex vivo* imaging of the lumbar spinal cord, a Nikon AX R MP Upright Multiphoton Microscope (Nikon Instruments Inc., Japan) was employed. Dissected lumbar spinal cord samples were placed in phosphate-buffered saline and imaged at room temperature. Excitation was performed at 820 nm, with fluorescence emission collected in the 506-594 nm range. Z-stack imaging was utilized to reconstruct three-dimensional distributions of UCNPs within the tissue samples.

Supporting Figures

Excitation Power Dependence of UCL Intensity

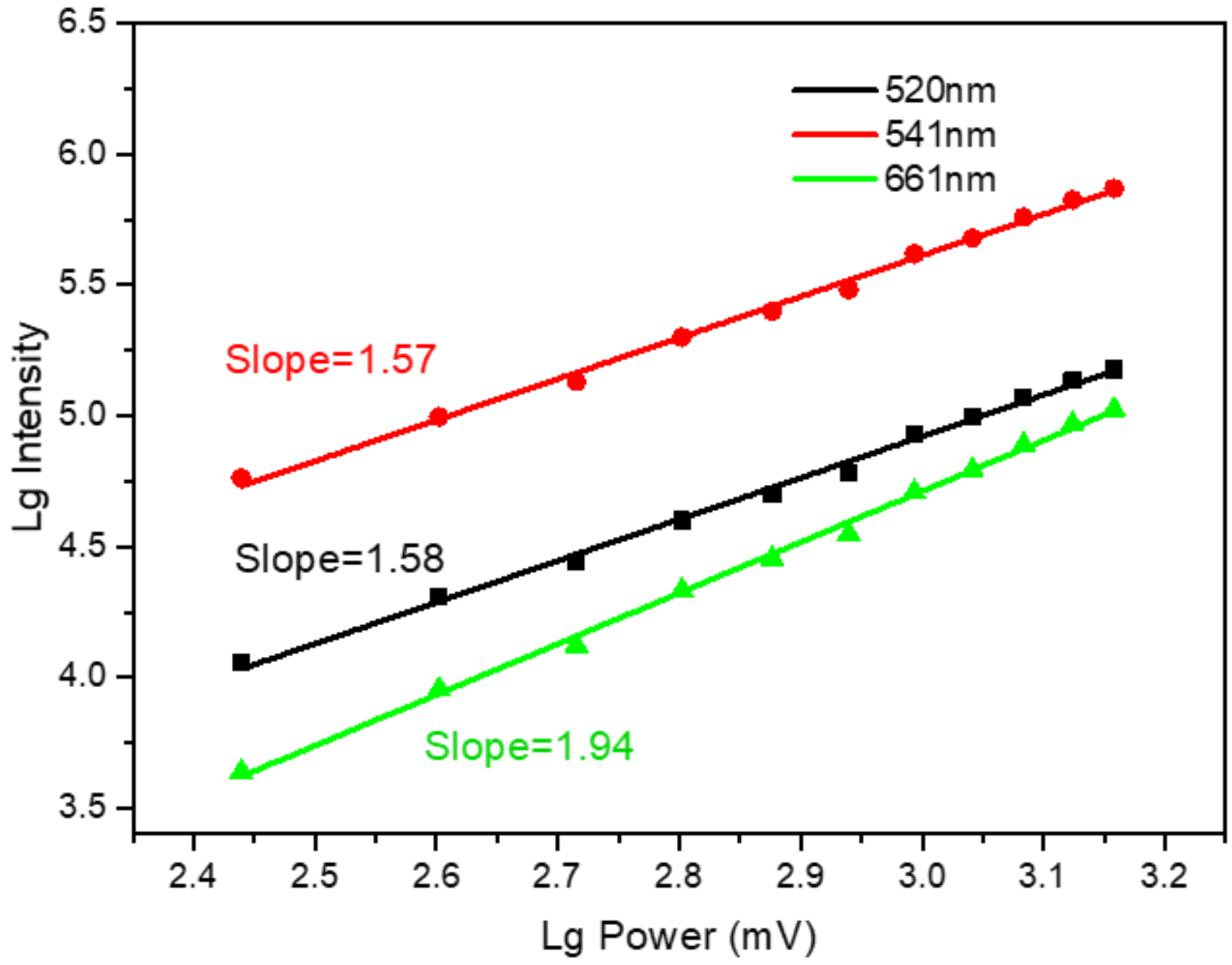

Figure S1. Intensity dependence of upconversion luminescence on the pump power in $NaYF_4$:YbEr@$NaYF_4$:YbNd@$NaYF_4$.

XRD of the Synthesized UCNPs

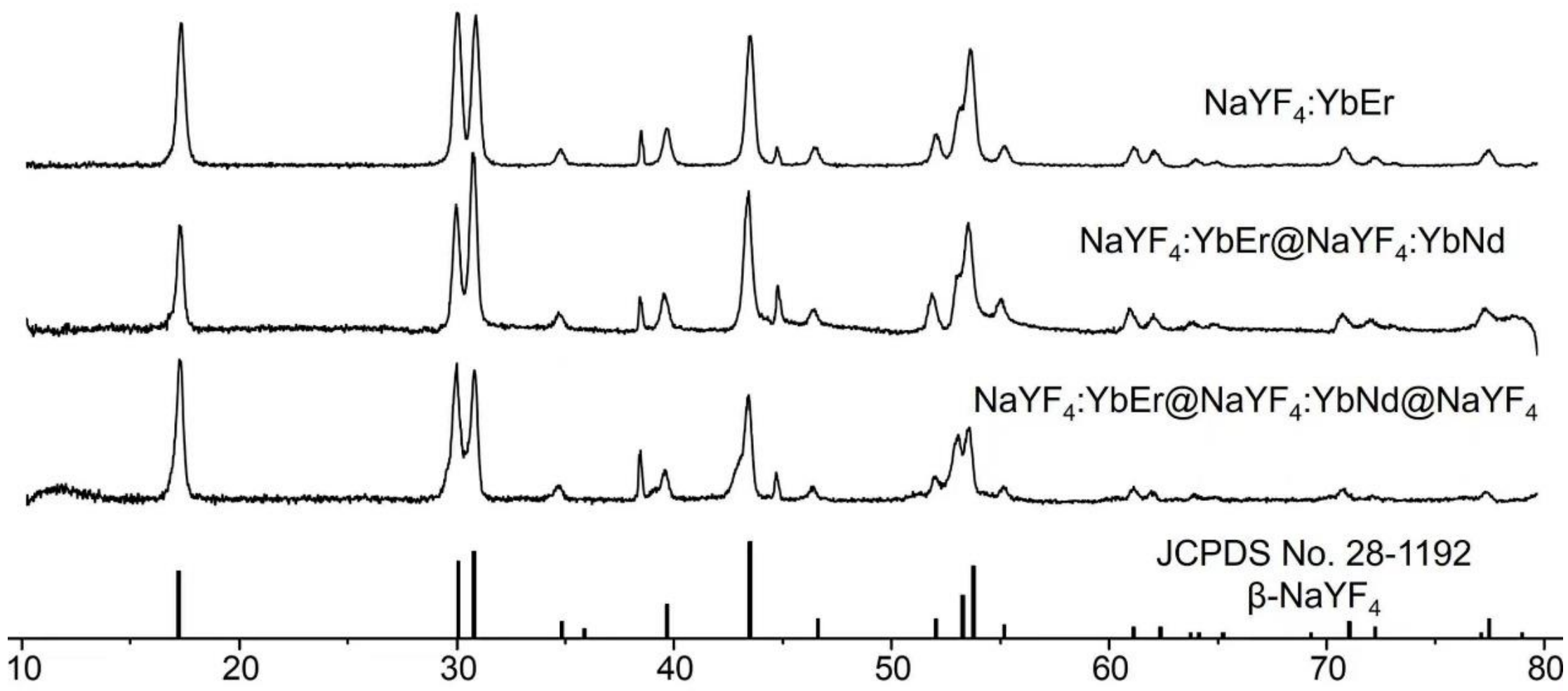

Figure S2. X-ray diffraction (XRD) patterns of the synthesized UCNPs.

Cytotoxicity of CSS-PEG-RVG29

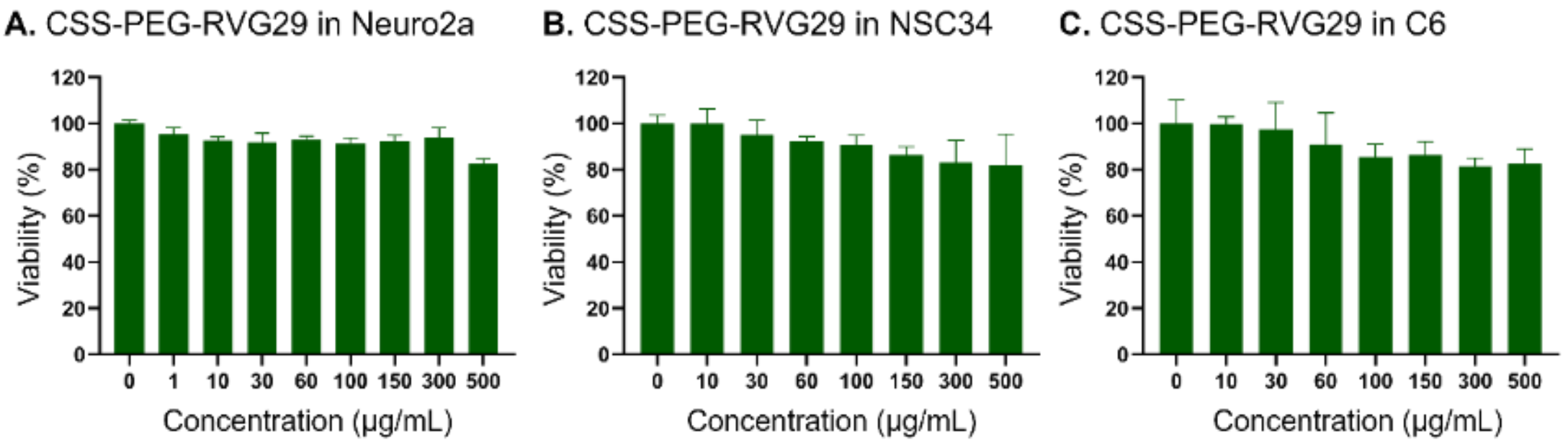

Figure S3. Cytotoxicity of CSS UCNPs. (A-C) Cytotoxicity of CSS-PEG-RVG29 at various concentrations in Neuro2a (A), NSC34 (B), and C6 cells (C) after 24 h incubation, measured by MTT assay.

Long-term Culture and Immunostaining of Primary Spinal Neurons

Long-term culture of primary spinal neurons

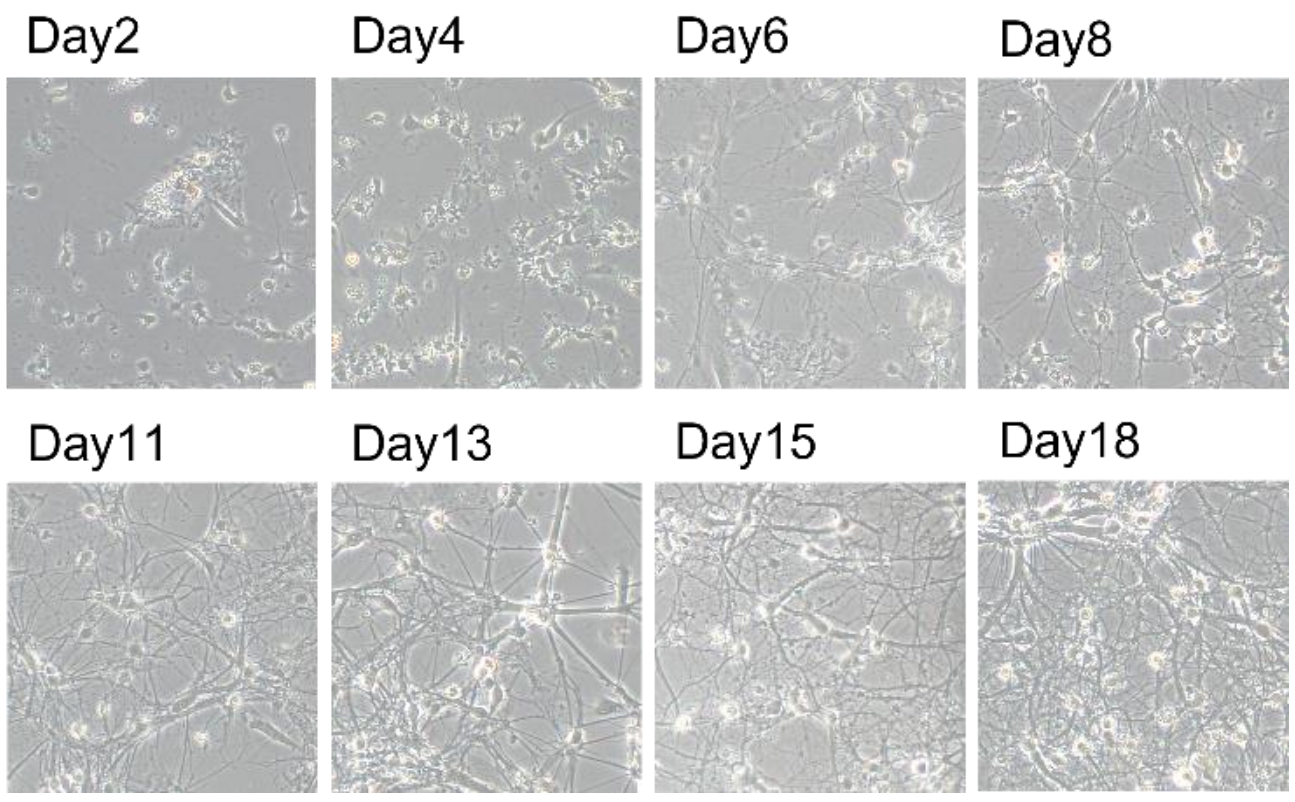

Figure S4. Long-term culture of primary spinal neurons from Day 2 to Day 18.

Immunostaining of primary spinal neurons

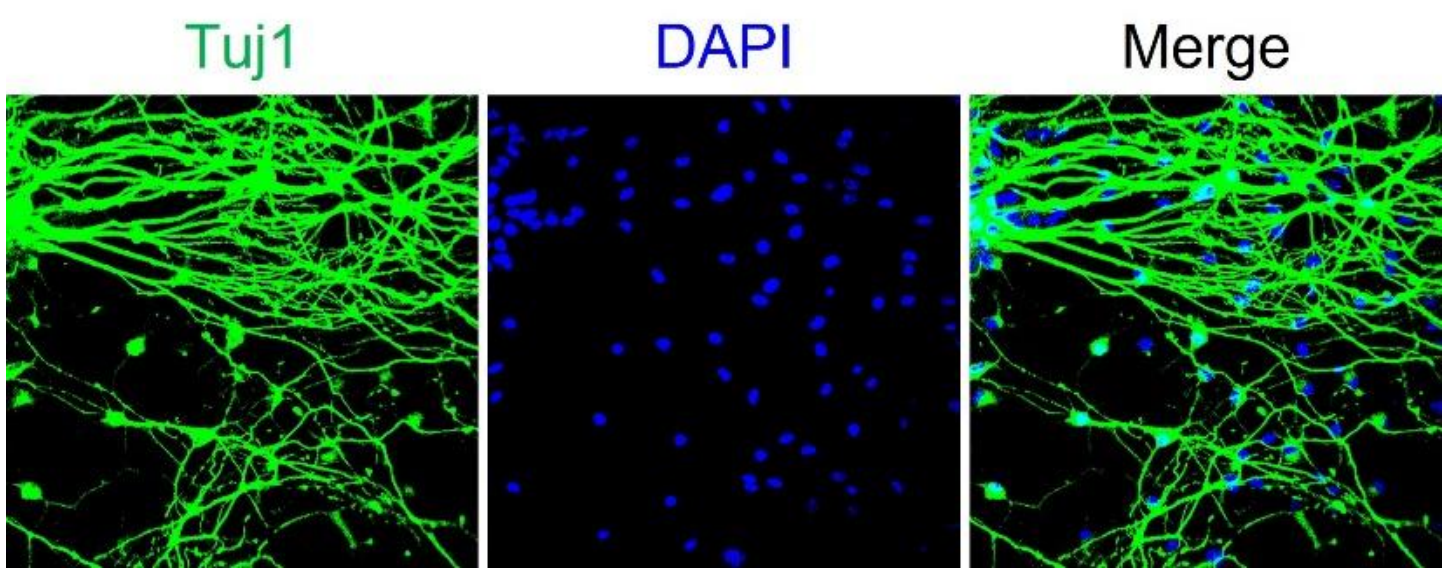

Figure S5. Primary spinal neurons were positive for Tuj1 (neuronal marker).

Long-term Culture and Immunostaining of Primary Cortical Neurons

Long-term culture of primary cortical neurons

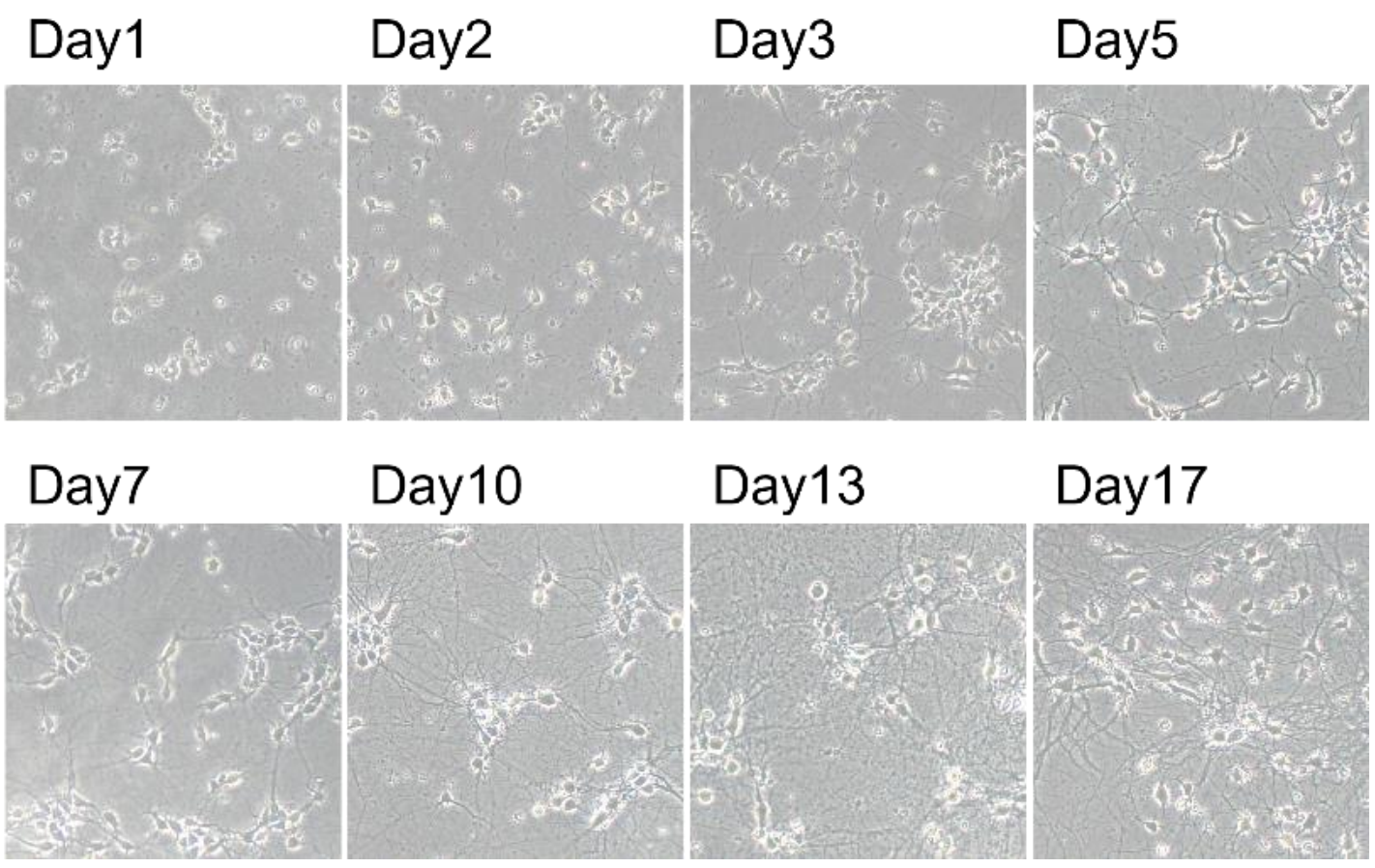

Figure S6. Long-term culture of primary cortical neurons from Day 1 to Day 17.

Immunostaining of primary cortical neurons

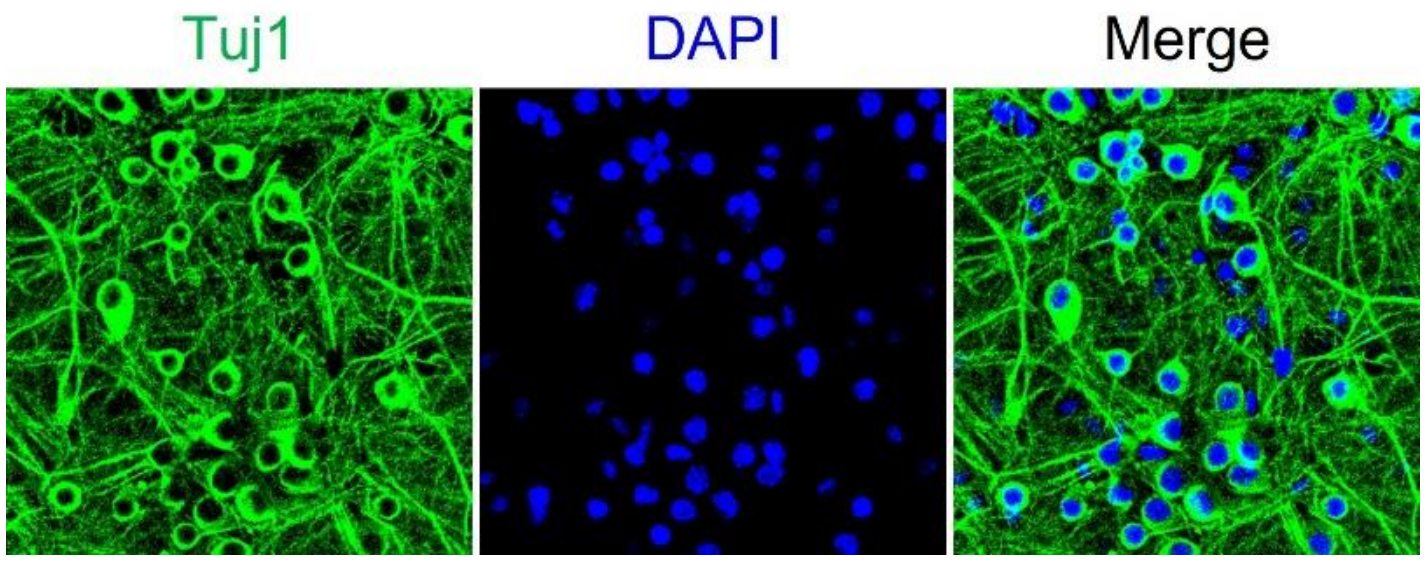

Figure S7. Primary cortical neurons were positive for Tuj1 (neuronal marker).

Contralateral Sciatic Nerve Imaging of UCNPs

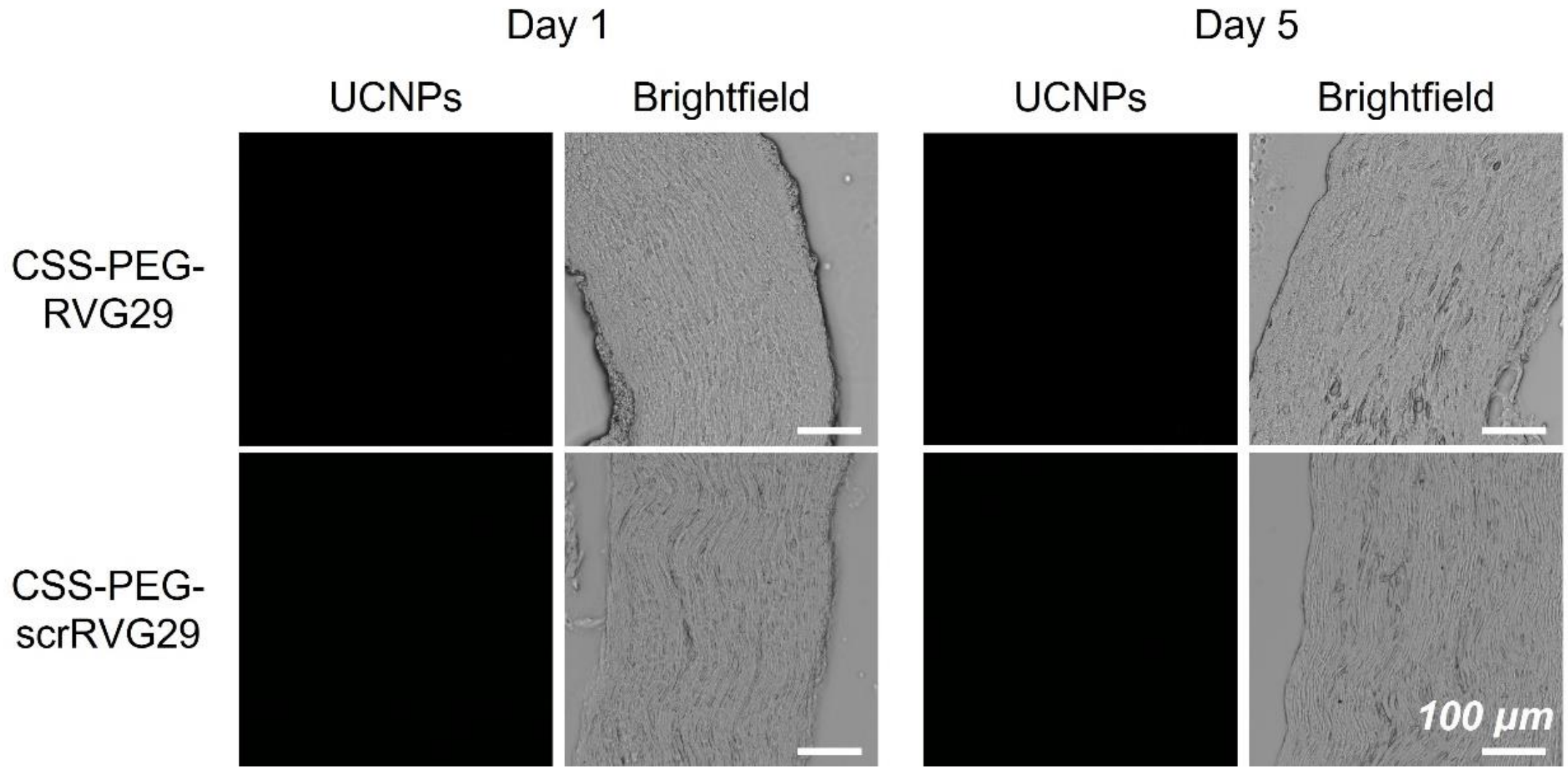

Figure S8. Confocal imaging of left (contralateral to the injection site) sciatic nerve tissue slices at Days 1 and 5 post-injection, injected with 2 μL of CSS-PEG-RVG29 or CSS-PEG-scrRVG29 solution (2 mg/mL). Both CSS-PEG-RVG29 and CSS-PEG-scrRVG29 cannot be transported to the contralateral sciatic nerves at Days 1 and 5 post-injection.

Fluorescence of CSS-PEG-RVG29 in Spleen, Liver, and Kidney

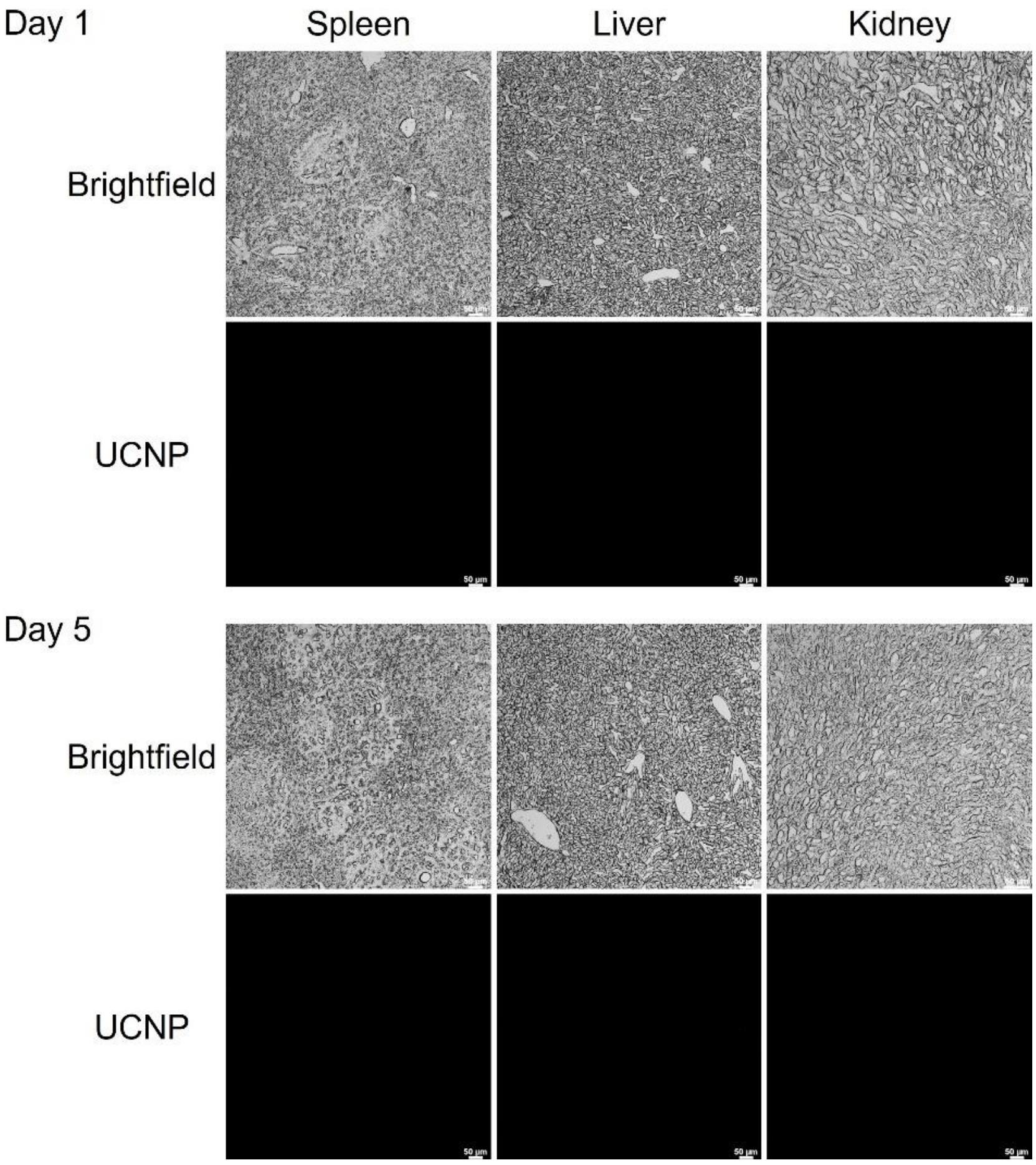

Figure S9. Fluorescence of CSS-PEG-RVG29 in mouse spleen, liver, and kidney at Days 1 and 5 post-injections.
